# Ubc9‐Mediated SUMOylation of RPL3, an Unappreciated Mechanism against Hepatocyte Senescence by Repressing the DHX9‐p16 Axis

**DOI:** 10.1002/advs.202510240

**Published:** 2025-10-20

**Authors:** Hao Xie, Zhichao Gao, Xin Liu, Shiyi Zhang, Yuhan Wang, Jia Gao, Ping Qi, Lu Zhang, Jiawei Zhao, Tian Xiong, Teng Huang, Jia Song, Qilin Yu, Shu Zhang, Yanjun Liu, Ping Yang, Maryam S. Al‐Motawa, Quan Gong, Junfeng Dong, Hao Yin, Fei Sun, Shiwei Liu, Cong‐Yi Wang

**Affiliations:** ^1^ Department of Respiratory and Critical Care Medicine the Center for Biomedical Research NHC Key Laboratory of Respiratory Diseases Tongji Hospital Tongji Medical College Huazhong University of Science and Technology Wuhan 430030 China; ^2^ Department of Clinical Laboratory Institute of Translational Medicine Renmin Hospital of Wuhan University Wuhan Hubei 430060 China; ^3^ Department of Interventional Radiology Renmin Hospital of Wuhan University Wuhan 430060 China; ^4^ Department of Geriatrics Tongji Hospital Tongji Medical College Huazhong University of Science and Technology Wuhan 430030 China; ^5^ Reproductive Medicine Center Tongji Hospital Tongji Medical College Huazhong University of Science and Technology Wuhan Hubei 430030 China; ^6^ The Center for Obesity and Metabolic Health Affiliated Hospital of Southwest Jiaotong University the Third People's Hospital of Chengdu 82 Qinglong Road Chengdu Sichuan 610031 China; ^7^ Shanxi Bethune Hospital Shanxi Academy of Medical Science Tongji Shanxi Hospital Third Hospital of Shanxi Medical University the Key Laboratory of Endocrine and Metabolic Diseases of Shanxi Province Taiyuan 030032 China; ^8^ Diabetes Research Center Qatar Biomedical Research Institute (QBRI) Hamad Bin Khalifa University (HBKU) Doha 34110 Qatar; ^9^ Department of Immunology School of Medicine Yangtze University Jingzhou Hubei 434023 China; ^10^ Organ Transplant Center Shanghai Changzheng Hospital (Second Affiliated Hospital of Naval Medical University) Shanghai 200003 China; ^11^ The Center for Biomedical Research Tongji Hospital Research Building Tongji Hospital Wuhan 430030 China

**Keywords:** aging, DHX9, metabolic dysfunction‐associated steatotic liver disease (MASLD), ribosomal protein L3 (RPL3), SUMOylation

## Abstract

Although Ubc9‐mediated SUMOylation are recognized to regulate the multiple aspects of hepatic biological processes, its impact on hepatic senescence and metabolic dysfunction‐associated steatotic liver disease (MASLD), however, is yet to be fully addressed. Herein noted an age‐dependent decrease of hepatic Ubc9 expression is first noted along with an escalated decrease of protein SUMOylation, which is coupled with enhanced senescent marker expressions both in humans and mice. Interestingly, Ubc9 is dispensable for liver development at the embryonic stage. However, Ubc9 deficiency in hepatocytes rendered mice with an exacerbated hepatic aging phenotype and more susceptible to fatty liver disease and steatohepatitis following the challenge of a methionine‐ and choline‐deficient (MCD)‐diet. Ii is further demonstrated that nuclear ribosomal protein L3 (RPL3) interacts with DExD/H‐box (DDX/DHX) helicases (DHX9), which then recruits RNA polymerase II to the p16 promoter to transcribe its expression, thereby exacerbating the hepatocyte aging process. However, Ubc9‐mediated SUMOylation prevents RPL3 nuclear translocation, by which it represses the expression of senescent markers such as p16 to attenuate the hepatic aging process. Together, the study highlights that Ubc9‐mediated SUMOylation of RPL3 could be an unappreciated mechanism against hepatic aging in clinical settings.

## Graphical abstract

Liver aging is characterized by a decline in the expression of the SUMO‐conjugating enzyme Ubc9, resulting in reduced SUMOylation levels in hepatocytes, particularly in the case of ribosomal protein RPL3. Disruption of RPL3 SUMOylation increases its nuclear translocation. Interestingly, ribosome‐free RPL3 facilitates the recruitment of helicase DHX9 to the promoter region of p16 within the nuclear, subsequently engaging RNA Pol II and enhancing the expression of the aging trigger factor p16. Consequently, targeting Ubc9 holds promise as a therapeutic strategy for treating liver aging in clinical settings.

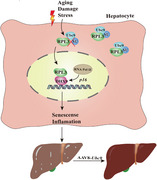

## 1 Introduction

The physiological aging process is an inevitable part of the life course for every mammalian species. However, exposure to environmental factors and lifestyle changes can induce an altered aging process, predisposing to the development of aging‐related diseases. Cellular senescence is a stable growth arrest that prevents hepatocytes from proliferation and differentiation.^[^ ^1^ ^]^ The decline in liver regenerative capacity heightens the susceptibility to various types of chronic liver diseases, including metabolic dysfunction‐associated steatotic liver disease (MASLD), steatohepatitis, cirrhosis, and hepatocellular carcinoma.^[^ ^2^, ^3^ ^]^ Actually, senescent hepatocytes drive age‐related disorders not only due to their dysfunction but also production of a bioactive secretome known as the senescence‐associated secretory phenotype (SASP).^[^ ^4^ ^]^ All of which diminishes hepatocyte plasticity, spelling failure to repair liver damage during the aging process. Accumulating evidence suggests that the typical senescence triggers are the induction of cell cycle inhibitors (e.g., p16^Ink4a^, p19^Arf^, and p21).^[^ ^5^, ^6^ ^]^ Studies also suggest that reduced expression of human hepatocyte nuclear factor 4 alpha (HNF4α) in a p53‐dependent manner significantly impairs hepatocyte development.^[^ ^7^ ^]^ While how such detailed machinery accounts for hepatocyte senescence and liver dysfunction remains elusive.

SUMOylation is an evolutionarily conserved post‐translational modification, in which the small ubiquitin‐like modifiers (SUMOs) covalently conjugate to the lysine (K) residue(s) in the consensus sequence (ψKXE) of a target protein.^[^ ^8^, ^9^ ^]^ SUMOylation requires ATP, typically with the assistance of three enzymes: a heterodimeric SUMO‐activating enzyme (E1), a sole SUMO‐conjugating enzyme (E2, Ubc9), and one of the several SUMO ligases (E3s).^[^ ^10^ ^]^ Importantly, SUMOylation is a reversible and highly dynamic process due to the existence of the sentrin‐specific protease (SENP) system.^[^ ^11^, ^12^, ^13^, ^14^ ^]^ The balance of orchestrated protein SUMOylation between deSUMOylation is essential for cellular biological processes and homeostasis in response to environmental changes or pathological insults.^[^ ^15^ ^]^ Therefore, SUMOylation has now received remarkable attention in regulating diverse cellular processes, including cell growth, apoptosis, and senescence.^[^ ^16^, ^17^, ^18^, ^19^, ^20^ ^]^

SUMOylation has previously been investigated in multiple aspects of liver biology, which displays a different alteration varying greatly from functional homeostasis to disease. For instance, SUMOylation of CCAAT/enhancer‐binding protein (C/EBPα) represses liver‐specific gene expression by ameliorating its interaction with BRG1, a core subunit of the SWI/SNF chromatin remodeling complex.^[^ ^21^, ^22^ ^]^ Moreover, the level of SUMOylated C/EBPα gradually decreases during the primary fetal hepatocyte differentiation.^[^ ^21^ ^]^ In line with this observation, the levels of SUMO1 and SUMO2 SUMOylated proteins are decreased in hepatocytes differentiated from human embryonic stem cells (hESCs).^[^ ^23^ ^]^ However, the detailed regulatory mechanisms of SUMOylation in liver function are yet to be fully elucidated. Herein, in this report, we demonstrated that Ubc9 is dispensable for liver development at the embryonic stage. However, *Ubc9* deficiency in hepatocytes rendered mice with an exacerbated liver dysfunction during the aging process and more susceptible to fatty liver disease and steatohepatitis following the challenge of an MCD‐diet. Mechanistically, we demonstrated that nuclear ribosomal protein L3 (RPL3) interacts with DExD/H‐box (DDX/DHX) helicases (DHX9), which then recruit RNA polymerase II to the *p16* promoter, thereby transcribing *p16* expression to initiate the aging process. However, Ubc9‐mediated SUMOylation of RPL3 prevents its nuclear translocation, by which SUMOylation attenuating *p16* expression to attenuate the hepatocyte aging process. Together, our study highlights that enhanced RPL3 SUMOylation could be an unappreciated mechanism against the hepatocyte aging process in clinical settings.

## 2 Results

### 2.1 Hepatocyte Senescence is Featured by the Attenuated SUMOylation Function

**Figure 1:**
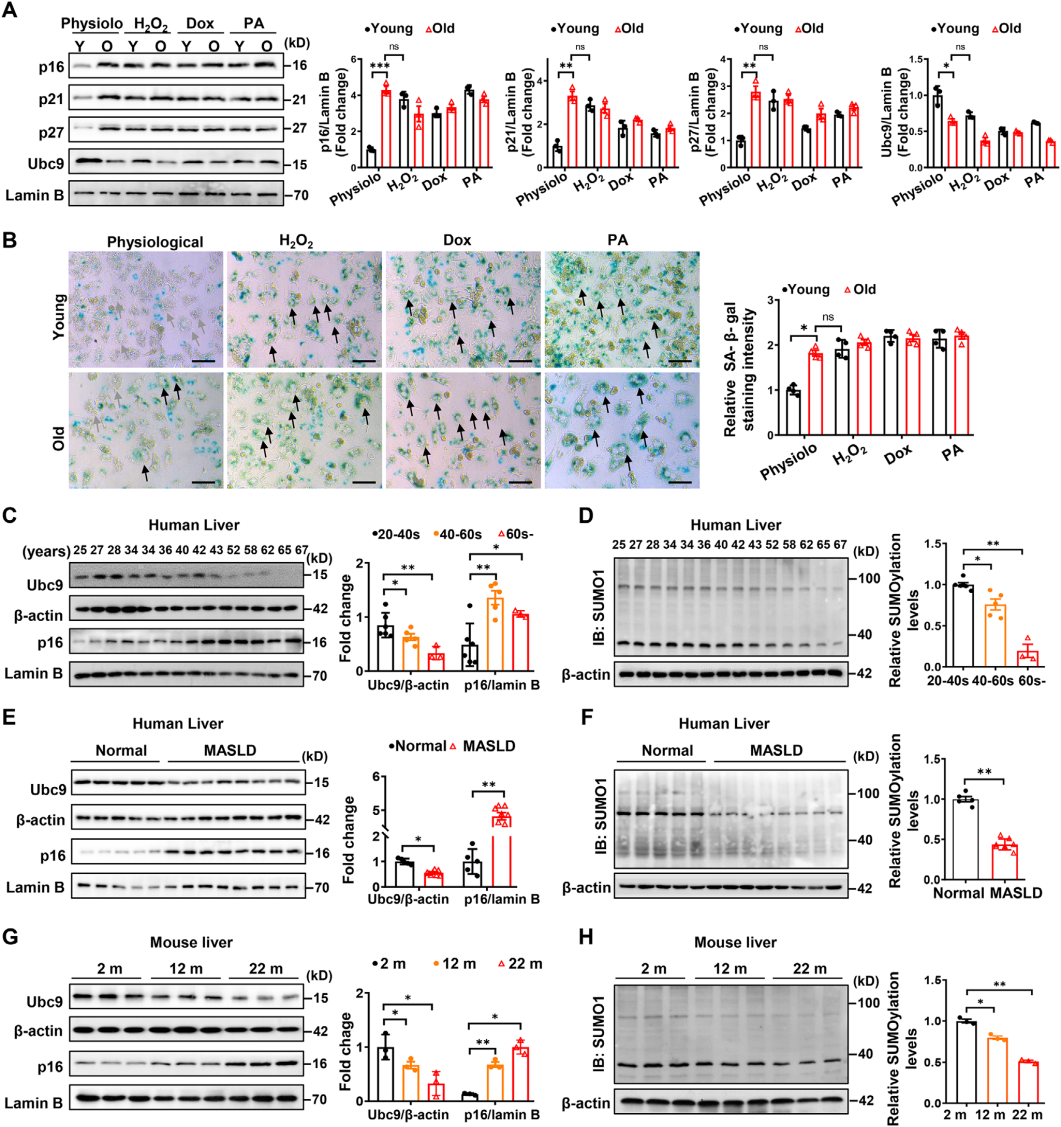
Ubc9 is downregulated in hepatocytes during the aging process. A) Hepatocytes isolated from young (2‐month‐old) and old (22‐month‐old) mice were subsequently treated with hydrogen peroxide (H_2_O_2_), doxorubicin (Dox), and palmitate (PA) to induce cellular stress and senescence. Cell lysates were harvested and analyzed for the expression of senescence markers p16、p21、p27 (*n* = 3). B) Representative β‐gal staining of each experimental group in (A). Gray arrows indicate hepatocytes with negative or weakly positive β‐gal staining, while black arrows highlight hepatocytes exhibiting typical strong positive β‐gal staining. Scale bars, 50 µm. C) Representative Western blot analysis of Ubc9 and senescent marker p16 protein expression in liver samples from humans of different ages (*n* = 14). D) Representative western blot analysis of SUMO1‐conjuncted proteins in liver tissue from individuals with different degrees of aging (*n* = 14). E, F) Representative western blot analysis of Ubc9, 16 protein expression (E), and SUMO1‐conjuncted substrates (F) in liver samples from patients with MASLD (*n* = 8) and control subjects (*n* = 5). G, H) Representative western blot analysis of Ubc9, p16 protein expression (E), and SUMO1‐conjuncted substrates (F) in liver samples from 2‐month, 12‐month, and 22‐month‐old mice (*n* = 3). All data are represented as mean ± SEM. One‐way ANOVA with Bonferroni post hoc analysis was used in (A), (C) (D) and (G), (H). The data in (E) and (F) were analyzed by an unpaired Student’ s *t*‐test. ^*^ *p* < 0.05, ^**^ *p* < 0.01.

To address the impact of Ubc9‐mediated SUMOylation on hepatocyte senescence, primary hepatocytes were isolated from young (2‐month‐old) and old (22‐month‐old) mice, followed by the challenge of 3 senescence inducers, hydrogen peroxide (H_2_O_2_), doxorubicin (Dox), and palmitic acid (PA), respectively. All 3 reagents markedly induced hepatocyte senescence, and the resulting aging phenotype was even stronger than that observed in 22‐month‐old hepatocytes (**Figure** **1**A). The senescent phenotype was also corroborated by the senescence‐associated β‐galactosidase (SA‐β‐gal) staining (Figure 1B).

Next, we first checked UBC9 expression in the livers from human subjects at different age stages. An age‐dependent decrease of UBC9 expression in the liver, along with a significantly induced expression of p16 (a senescent marker), was observed (Figure 1C). Consistently, an aging‐related progressive decrease of global SUMOylations was detected in the liver (Figure 1D). Studies in hepatic biopsy samples also revealed that MASLD patients were featured by the markedly downregulated UBC9 expression as compared to their counterparts, which was coupled with a markedly elevated p16 expression and a global reduction of SUMOylated proteins (Figure 1E,F). Similarly, liver tissues from 2‐, 12‐, and 22‐month‐old mice were manifested by the age‐dependent escalated decline of Ubc9 expression (Figure 1G) and protein SUMOylations (Figure 1H). Notably, this phenotypical change specifically occurred in hepatocytes (Figure S1A, Supporting Information), rather than in nonparenchymal cells (Figure S1B, Supporting Information). Indeed, either H_2_O_2_ or Dox dose‐dependently repressed Ubc9 expression in primary hepatocytes isolated from 2‐month‐old mice, which was also coupled with a significant reduction of global SUMOylated substrates (Figure S1C–F, Supporting Information). Together, these findings support that Ubc9‐mediated SUMOylation may play a pivotal role in regulating hepatocyte senescence.

### 2.2 *Ubc9* Deficiency Exacerbates Hepatocyte Senescence and Liver Dysfunction

**Figure 2:**
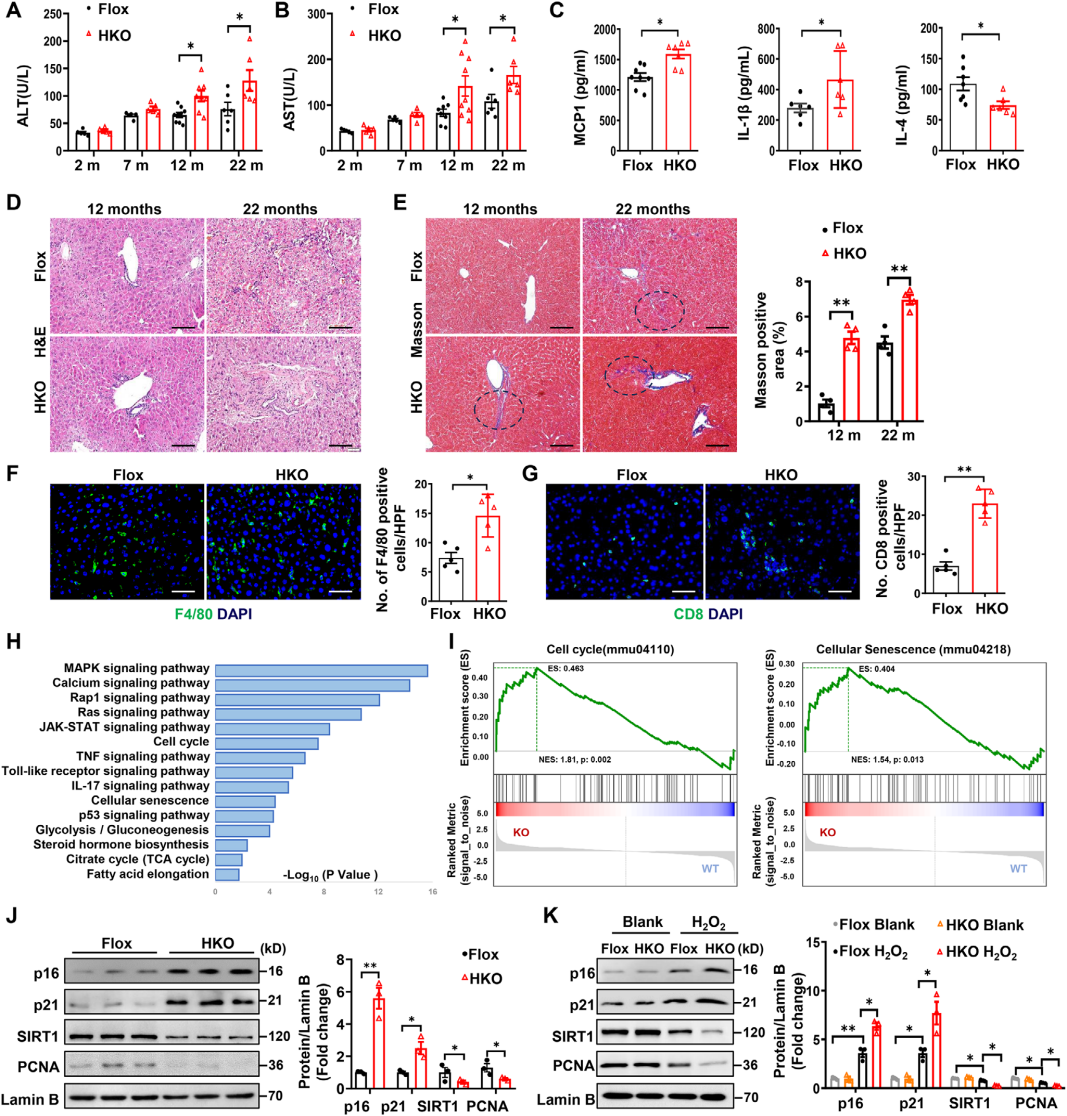
Hepatocyte‐specific *Ubc9* knockout exacerbates hepatic senescence. A, B) The serum levels of alanine aminotransferase (ALT) (A) and aspartate aminotransferase (AST) (B) of HKO and control mice at different periods. C) ELISA analysis of inflammatory factors MCP‐1, IL‐1β, and IL‐4 in the serum of 12‐month‐old HKO and Flox mice (*n* = 7 or 8). D, E) H&E and Masson staining of the liver sections of the mice in the indicated group. Circled regions showed prominent pericentral and perisinusoidal fibrosis (E). Scale bars, 100 µm. F) Representative immunofluorescence staining to assess CD8 positive T cell infiltration liver sections from 12‐month‐old HKO and Flox mice (*n* = 5). Scale bars, 50 µm. G) Representative immunofluorescence staining for F4/80 to assess total macrophage accumulation in liver sections from 12‐month‐old HKO and Flox mice (*n* = 5). Scale bars, 50 µm. H) The KEGG enrichment pathways in the transcriptomes of HKO mice compared with Flox. I) Gene Set Enrichment Analysis (GSEA) of senescence‐associated pathways in hepatocytes isolated from 12‐month‐old HKO and Flox mice. J) Representative western blot analysis of senescent and proliferative markers in liver samples of 12‐month‐old HKO and Flox mice (*n* = 3). K) Primary hepatocytes isolated from 2‐month‐old HKO or Flox mice were treated with hydrogen peroxide (H_2_O_2_,100 µm) for 6 h. Cell lysates were subjected to western blot analysis, and protein expression of p16, p21 SIRT1, and PCNA was determined (*n* = 3). All data are represented as mean ± SEM. The data in (A), (B), (C), and (E–G) were analyzed by unpaired Student’ s *t*‐test. One‐way ANOVA with Bonferroni post hoc analysis was used in (J) and (K). ^*^ *p* < 0.05, ^**^ *p* < 0.01.

To address the functional relevance of Ubc9‐mediated SUMOylation in hepatocyte senescence, we generated a hepatocyte‐specific *Ubc9* knockout (HKO) mice by crossing the *Ubc9^fl/fl^* mice with the *Alb‐Cre* mice, in which exons 2–4 of *Ubc9* were deleted through the Cre‐Loxp system.^[^ ^24^ ^]^ As expected, Ubc9 was dramatically decreased in the liver, but not in the kidney and heart (Figure S2A–C, Supporting Information), and particularly, Ubc9 was almost undetectable in hepatocytes isolated from the HKO mice (Figure S2D, Supporting Information). Deletion of *Ubc9* significantly reduced the total SUMO1‐conjugated proteins in the liver and abolished SUMOylation in hepatocytes (Figure S2E,F, Supporting Information). The HKO mice looked normal during their nursing period and did not show any developmental defect in the liver (Figure S2G, Supporting Information). However, an age‐dependent liver dysfunction was observed in HKO mice, as manifested by the progressive increase of alanine aminotransferase (ALT) (**Figure** **2**A) and aspartate aminotransferase (AST) levels (Figure 2B) under a normal chow diet (NCD). Based on the above results, 12‐month‐old HKO mice were next selected to further address the role of Ubc9 in hepatocyte senescence. Consistently, higher serum MCP‐1 and IL‐1β, along with lower IL‐4 levels, were detected in the HKO mice (Figure 2C). Additionally, the HKO mice only displayed a moderately higher fasting blood glucose level (Figure S2H, Supporting Information), but with a comparable postprandial blood glucose level (Figure S2I, Supporting Information). Histological analysis also demonstrated age‐dependent structural alterations, with notably more pronounced disruption of hepatic lobules and pericentral and perisinusoidal fibrosis observed in HKO mice at 12 months of age, which became even more severe by 22 months (Figure 2D,E; Figure S2J, Supporting Information). Moreover, the HKO mice exhibited significantly higher hepatic infiltration of F4/80^+^ macrophages (Figure 2F) and CD8^+^ T cells (Figure 2G), indicating a severe senescence‐associated secretory phenotype (SASP).

To evaluate the generalizability of our findings, we conducted transcriptomic sequencing analysis on hepatocytes derived from 12‐month‐old mice in both the HKO and Flox groups. The most significantly enriched KEGG pathways included MAPK signaling, cellular senescence, and metabolism pathways (Figure 2H). Furthermore, gene‐set enrichment analysis (GSEA) of the transcriptomic data confirmed that biological pathways related to cellular senescence and cell cycle were significantly activated in the HKO group (Figure 2I). Notably, livers from HKO mice exhibited enhanced expression of senescent markers such as p16 and p21, but repressed expression of proliferative markers such as SIRT1 and PCNA, at both protein (Figure 2J) and mRNA (Figure S2K, Supporting Information) levels. Additionally, analysis of Hepatic NAD+/NADH ratio (Figure S2L, Supporting Information) further confirmed higher severity of hepatic senescence in the HKO mice. Importantly, in *Ubc9*‐deficient hepatocytes from 2‐month mice, H_2_O_2_ or Dox treatment led to elevated p16 and p21 expression and reduced SIRT1 and PCNA expression (Figure 2K; Figure S2M, Supporting Information). This phenotypical change was coupled with a remarkably increased expression of γH2AX (Figure S2N, Supporting Information), a marker of DNA damage, supporting that Ubc9‐mediated SUMOylation likely maintains genome integrity, thereby protecting hepatocytes against cellular senescence.

### 2.3 *Ubc9* Deficiency Exacerbates MCD‐Induced Hepatic Steatosis and Senescence

**Figure 3:**
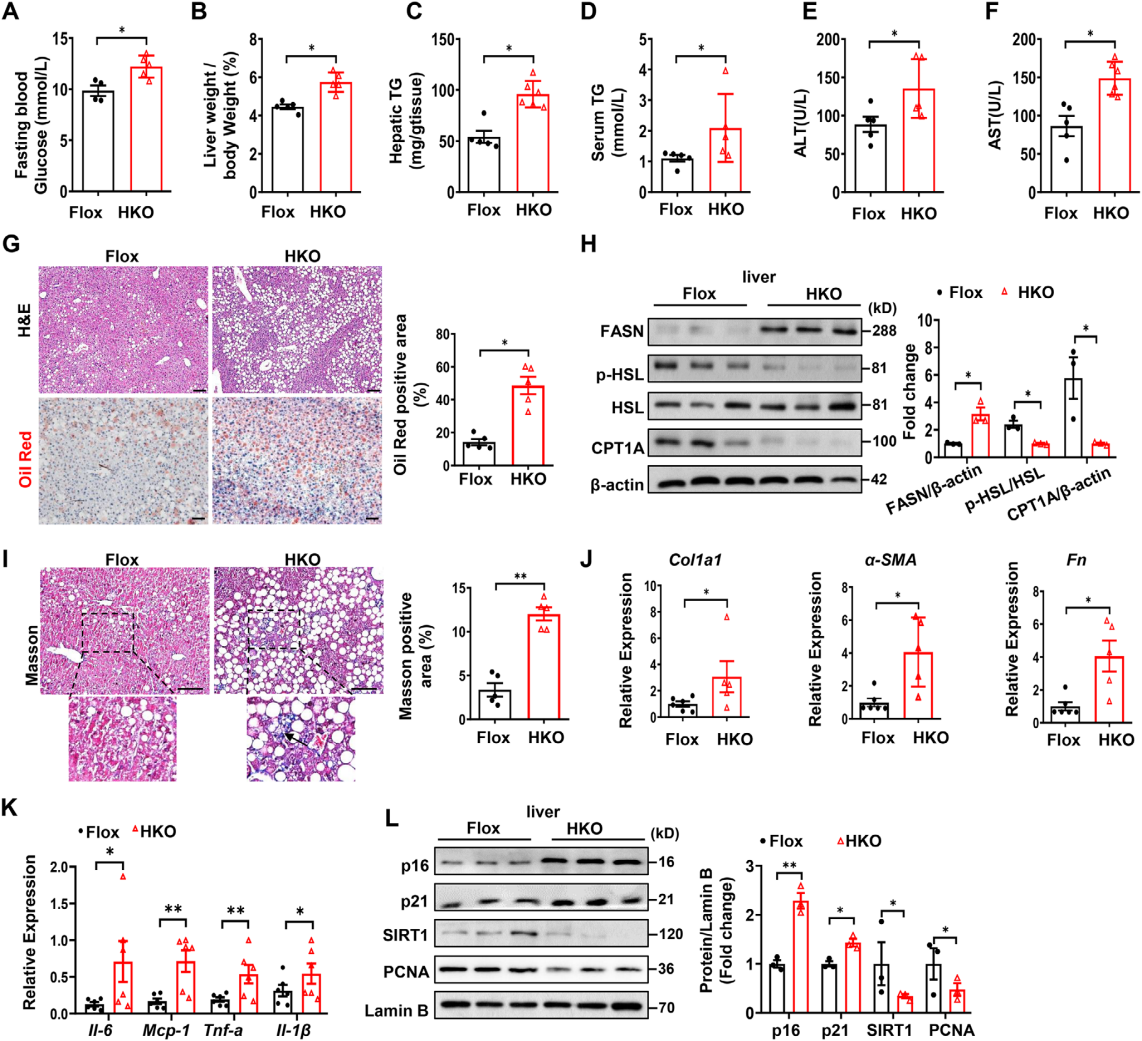
Hepatocyte‐specific *Ubc9* knockout exacerbates MCD diet‐induced hepatic steatosis, inflammation, and fibrosis. A, B) Fasting blood glucose (A) and ratios of liver weight to body weight (B) of HKO mice and their corresponding controls after MCD diet consumption for 6 weeks (*n* = 5 or 6). C, D) The hepatic (C) and serum (D) triglyceride contents of the mice in the indicated group (*n* = 5 or 6). E, F) The serum levels of alanine aminotransferase (ALT) (E), aspartate aminotransferase (AST) (F) of HKO and control mice after feeding a MCD diet for 8 weeks (n = 5 or 6). G) Representative HE (upper) and oil red O (lower) staining of the liver sections of the mice in the indicated group. Scale bars, 100 µm. H) Representative Western blot analysis of proteins (related to fatty acid metabolism) expression in the livers of the mice in the indicated group (*n* = 3). I) Masson staining in liver sections of HKO and control mice. The black arrow indicated perisinusoidal fibrosis. Scale bars, 100 µm. J, K) Relative mRNA levels of genes related to fibrosis (J) and inflammatory response (K) in the livers of the mice in the indicated group (*n* = 5 or 6). L) Representative western blot analysis for determining the protein expression of p16, p21, SIRT1 and PCNA (*n* = 3). All data are represented as mean ± SEM. The data in (A–L) were analyzed by unpaired Student’ s *t*‐test. ^*^ *p* < 0.05, ^**^ *p* < 0.01.

The above results prompted us to explore the role of Ubc9 in the pathogenesis of MASLD, in which 8‐week‐old HKO mice were challenged by the MCD diet for 6 weeks. The HKO mice were characterized by the higher fasting blood glucose levels (**Figure** **3**A), greater liver‐weight to body‐weight ratios (Figure 3B), and higher TG levels in the liver (Figure 3C) and serum (Figure 3D) following MCD insult. Impaired liver function was also noted, as evidenced by the increased ALT (Figure 3E) and AST levels (Figure 3F), along with an increased ectopic lipid deposition in the liver (Figure 3G). In line with these results, enhanced expression of lipogenic genes such as fatty acid synthase (FASN) along with decreased expression of lipolytic genes such as p‐HSL and CPT1A were noted (Figure 3H). *Ubc9* deficiency also resulted in enhanced hepatic perisinusoidal fibrosis, as determined by Masson staining (Figure 3I), and increased expression of fibrotic markers Collagen 1 (Col1a1), α‐SMA, and Fibronectin (Fn) (Figure 3J). Higher expression of pro‐inflammatory factors such as *Il‐6*, *Mcp‐1*, *Tnf‐α*, and *Il‐1β* was further noted in the HKO mice (Figure 3K). Particularly, the MCD diet significantly exacerbated hepatocyte senescence, as evidenced by the increased expression of p16 and p21, coupled with a reduced expression of proliferative markers (Figure 3L). Together, these data demonstrate that loss of *Ubc9* aggravates the progression of MASLD and senescence of hepatocytes.

Next, senolytics dasatinib and quercetin (D+Q) were employed to confirm the senescent phenotype mediated by *Ubc9* deficiency. For this purpose, 18‐month‐old HKO mice were supplemented with dasatinib and quercetin for 2 months as described (Figure S3A, Supporting Information). Depletion of senescent hepatocytes by dasatinib and quercetin completely abolished the differences in terms of serum ALT and AST levels (Figure S3B,C, Supporting Information), and the production of pro‐ and anti‐inflammatory (Figure S3D–F, Supporting Information) factors between the HKO and control mice. The senolytics also almost diminished the differences of hepatic structural changes (Figure S3G, Supporting Information), and the infiltration of macrophages and CD8 T cells (Figure S3H,I, Supporting Information), as well as the expression of senescent markers (Figure S3J, Supporting Information), between the HKO and control mice. To delve deeper into the effects of senescent hepatocyte accumulation and senolytic intervention in HKO mice, we isolated hepatocytes from 2‐month‐old HKO and control mice, induced senescence with H_2_O_2_ or Dox, and treated them with D+Q. Transcriptomic profiling showed that HKO‐derived hepatocytes exhibited high severity of senescence phenotypes following H_2_O_2_ or Dox treatment, along with significantly dysregulated key pathways (Figure S3K–N, Supporting Information). Comparative analysis between vehicle‐ and D+Q‐treated Dox‐ or H_2_O_2_‐induced HKO hepatocytes demonstrated that senolytic intervention not only suppressed expression of senescence‐associated inflammatory genes but also improved drug and bile acid metabolism, fatty acid metabolism, and xenobiotic detoxification (Figure S3O,P, Supporting Information). Collectively, our findings support that Ubc9‐mediated SUMOylation plays an indispensable role for hepatocytes against senescence.

### 2.4 Ubc9 Mediates RPL3 SUMOylation in Hepatocytes during the Aging Process

**Figure 4:**
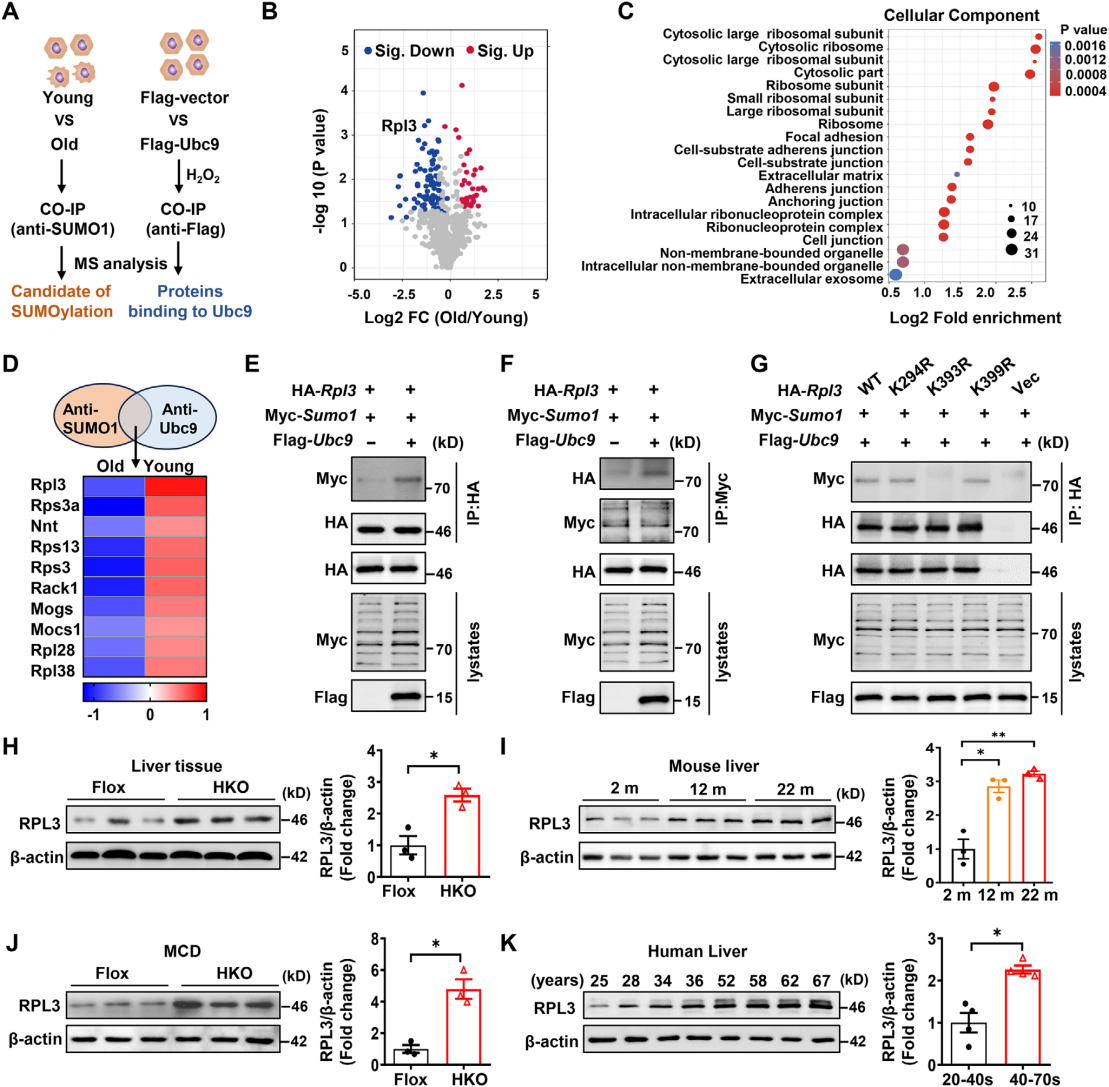
RPL3 is a novel SUMOylation target. A) A brief schematic diagram showing the procedure of identifying the downstream targets regulated by Ubc9‐mediated SUMOylation. B) A volcano plot showing DEGs (red, upregulated genes; blue, downregulated genes) in young or old mouse primary hepatocytes. C) GO enrichment‐based clustering was applied to analyze the potential SUMOylated proteins molecular functions. D) Heatmap indicating the 10 overlapped proteins (*p* < 0.05) between SUMO1‐bound proteins and proteins that interacted with Ubc9. E, F) Representative coimmunoprecipitation (co‐IP) assays were performed to examine the SUMOylation of RPL3 in AML12 cells co‐transfected with HA‐*Rpl3* WT, Myc‐*SUMO1*, or Flag‐*Ubc9* adenovirus. Empty vectors were used as a control. G) In screening for potential SUMOylated lysine(s) of RPL3, HEK293T cells were transfected with Flag‐SUMO1 and either HA‐*Rpl3* WT or the K294R, K393R, K399R mutant of HA‐Rpl3. Cell lysate was harvested in the presence of 10 mM NEM, and immunoprecipitated HA‐RPL3 was probed for SUMOylation using an anti‐HA antibody. H, I) Representative Western blot analysis for protein expression of RPL3 in liver homogenates from 12‐month‐old HKO and control mice (*n* = 3) (H) and from 2‐month, 12‐month, and 22‐month‐old mice (*n* = 3). (I). J, K) Representative western blot analysis of RPL3 in liver tissue from HKO and control mice fed with MCD diet for 8 weeks (*n* = 3) (J) and individuals with different degrees of aging (K). All data are represented as mean ± SEM. The data in (H), (J) ANwere analyzed by unpaired Student’ s *t*‐test. One‐way ANOVA with Bonferroni post hoc analysis was used in (I). ^*^ *p* < 0.05, ^**^ *p* < 0.01.

To elucidate the underlying mechanisms by which Ubc9‐mediated SUMOylation regulates hepatocyte senescence, we first intend to identify the critical SUMOylated target(s) relevant to the aging process. To this end, primary hepatocyte lysates originated from young (2‐month‐old) and aged (22‐month‐old) mice were subjected to co‐immunoprecipitation (Co‐IP) using an anti‐SUMO1 polyclonal antibody, and the resulting precipitates were analyzed by mass‐spectrometry (MS). We also conducted Co‐IP/MS analysis using Ubc9 overexpressed AML12 cells treated with H_2_O_2_ to pull‐down Ubc9 conjugated proteins (**Figure** **4**A). Quantitative analysis of SUMOylated proteins identified 72 downregulated substrates and 32 upregulated targets in young mice as compared to those of aged mice (Figure 4B). Gene Ontology (GO) analysis demonstrated that proteins associated with the ribosome signaling pathway possessed the highest enrichment score (Figure 4C). Eighteen SUMOylated candidates were identified in the interactome analysis, and 10 of them were also present in the studies conducted in AML12 cells. Particularly, RPL3, a component of the large ribosomal subunit (it also exerts a variety of extra‐ribosomal functions),^[^ ^25^, ^26^ ^]^ ranked the top one within the list of proteins according to the abundance of protein, fold change, and significance (Figure 4D).

To confirm that Ubc9 mediates SUMOylation of RPL3, AML12 cells were transduced with adenoviruses carrying HA‐*Rpl3*, Flag‐*Ubc9*, and Myc‐*SUMO1*. Co‐IP assays indeed detected SUMOylated form of RPL3 in cells overexpressing *Ubc9* (Figure 4E,F). SUMOplot analysis of RPL3 identified 3 potential SUMOylation sites at lysine(K)294, 393, and 399, and all of these lysine residues were evolutionarily conserved (Figure S4A, Supporting Information). Next, each of these lysine residues was mutated to arginine to identify the exact SUMOylation site(s). Interestingly, mutation at K393 completely abolished RPL3 SUMOylation, while the SUMOylated RPL3 could still be detected in cells transduced with adenoviruses carrying the K294R and K399R mutants (Figure 4G), indicating that K393 is the only SUMOylation site.

It was noted that *Ubc9* deficiency resulted in elevated RPL3 protein levels in the livers of 12‐month‐old HKO mice (Figure 4H). Similarly, H_2_O_2_ challenge also increased RPL3 protein levels in primary hepatocytes isolated from 2‐month‐old HKO mice (Figure S4B, Supporting Information). An age‐dependent RPL3 upregulation was detected in mouse livers from different age groups (Figure 4I), and consistently, H_2_O_2_ induced RPL3 expression in hepatocytes isolated from 2‐month‐old mice in a dose‐dependent manner (Figure S4C, Supporting Information). Moreover, challenge of HKO mice with an MCD diet induced an increase in RPL3 expression in the livers (Figure 4J). Interestingly, a similar trend in terms of enhanced RPL3 expression with the aging process was also observed in human livers (Figure 4K). In contrast, the levels of SUMOylated RPL3 were found to be lower in livers from patients with MASLD compared to those from control subjects (Figure S4D, Supporting Information). However, disruption of RPL3 SUMOylation did not impact its stability (Figure S4E, Supporting Information), which was also confirmed in *Ubc9*‐deficient hepatocytes in the presence of cycloheximide (CHX) (Figure S4F, Supporting Information), and comparable ubiquitylated levels for *Rpl3* WT and K393R in the presence of a proteasome inhibitor MG132 (Figure S4G, Supporting Information). These results suggest that the increased RPL3 expression is directly induced by the aging process rather than by the enhancement of its stability through SUMOylation.

### 2.5 Loss of RPL3 SUMOylation Exacerbates Hepatocyte Senescence and Liver Dysfunction

**Figure 5:**
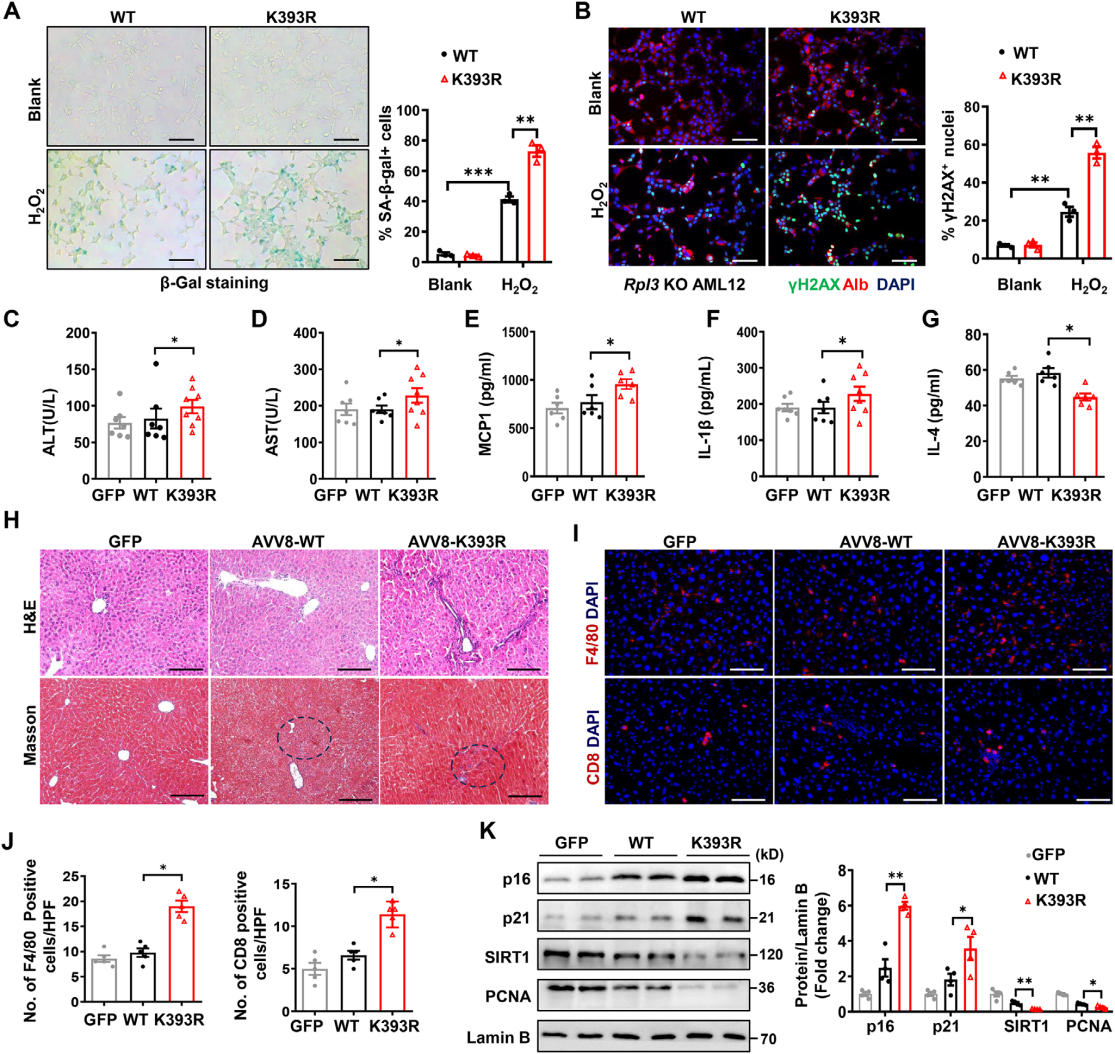
RPL3 SUMOylation deficiency exacerbates hepatocyte senescence and liver dysfunction. A, B) Representative SA‐b‐gal staining images (A) and representative images of immunofluorescence staining (B) of *Rpl3* CRISPR KO AML‐12 cells infected by Rpl3 WT or K393R adenovirus. Cells were stained after H_2_O_2_ (100 µm) induced senescence (*n* = 4). Scale bars, 50 µm. C, D) 18‐month‐old mice were injected with AAV‐GFP or AAV‐*Rpl3* WT or AAV‐*Rpl3* K393R. After 4 months, analysis of the serum levels of ALT (C) and AST (D) in mice from the indicated groups (*n* = 5–7). E–G) Serum levels of MCP‐1(E), IL‐1β (F), and IL‐4 (G) in mice from the indicated groups (*n* = 5–7). H) HE and masson staining of liver sections of the indicated groups of mice. Circled regions showed mild perisinusoidal fibrosis. Scale bars, 100 µm. I, J) Representative immunofluorescence staining images of F4/80 and CD8 (I) of the liver sections of the indicated groups of mice, along with corresponding quantitative analysis (J) (*n* = 5). Scale bars, 50 µm. K) Western blot analysis of p16, p21, SIRT1, and PCNA in liver samples of the indicated groups of mice (*n* = 3). All data are represented as mean ± SEM. One‐way ANOVA with Bonferroni post hoc used for data analysis. ^*^ *p* < 0.05, ^**^ *p* < 0.01. ^***^ *p* < 0.001.

To address the functional relevance of RPL3 SUMOylation in hepatocyte senescence, we established an *Rpl3* knockout AML12 (KO) cell line using the CRISPR/Cas9 system. Given that both free fatty acids (FFA) and reactive oxygen species (ROS) play pivotal roles in inducing hepatocyte aging and lipid metabolism disorders, with ROS acting as the immediate trigger,^[^ ^27^, ^28^ ^]^ we adopted a hydrogen peroxide‐induced model to explore the intricate role of RPL3 SUMOylation in regulating the process of hepatic senescence. H_2_O_2_ dose‐dependently induced Rpl3 expression in the WT cells but not in the KO cells (Figure S5A, Supporting Information). The KO cells were next induced for stable ectopic *Rpl3* WT or K393R expression through adenoviral transduction (Figure S5B, Supporting Information). Upon H_2_O_2_ exposure, the *Rpl3*‐K393R transduced cells exhibited a marked increase in p16 and p21 expression and a decrease in SIRT1 and PCNA levels compared to those of *Rpl3*‐WT transduced cells (Figure S5C, Supporting Information). Immunostaining also confirmed enhanced senescence‐associated beta‐galactosidase (SA‐β‐gal) expression in the *Rpl3*‐K393R transduced *Rpl3* KO hepatocytes (**Figure** **5**A). Indeed, the intensity of γH2AX expression was significantly higher in *Rpl3*‐K393R transduced *Rpl3* KO hepatocytes (Figure 5B).

To provide more comprehensive evidence of Rpl3 SUMOylation in the pathogenesis of hepatic senescence, the adeno‐associated virus 8 (AAV8) particles carrying *Rpl3* WT (AAV8‐*Rpl3*‐WT) or K393R (AAV8‐*Rpl3*‐K393R) or AAV8‐GFP were administrated into the 18‐month‐old mice via tail vein, and the mice in each group were fed with a normal chow diet absence of any intervention (Figure S5D, Supporting Information). Western blotting confirmed hepatic *Rpl3*‐WT or ‐K393R overexpression as compared to the control mice (AAV8‐GFP) (Figure S5E, Supporting Information). All mice were subjected to analysis of hepatic injury or dysfunction following 4‐month of injection. Notably, AAV8‐Rpl3‐K393R mice exhibited elevated serum levels of ALT (Figure 5C) and AST (Figure 5D), along with higher circulating Mcp‐1 and IL‐1β, but lower IL‐4 (Figure 5E–G). Histological analysis revealed a higher severity of liver injury in Rpl3‐K393R‐transduced mice, characterized by the disrupted hepatic lobular architecture, a marked aggregation of inflammatory cells, and pronounced liver fibrosis (Figure 5H; Figure S5G, Supporting Information). These pathological changes were coupled with an increased infiltration of macrophages and CD8 T cells (Figure 5I,J). Consistent with these findings, pro‐inflammatory cytokine mRNA levels were upregulated in the livers of AAV*‐Rpl3*‐K393R transduced mice (Figure S5F, Supporting Information). Furthermore, heightened expression of p16 and p21, along with reduced levels of SIRT1 and PCNA, was detected in the livers from mice transduced with AAV‐Rpl3‐K393R (Figure 5K). Taken together, these data support that disruption of Rpl3 SUMOylation accelerates hepatic injury and dysfunction by enhancing hepatocyte senescence.

### 2.6 SUMOylation Regulates RPL3 Nuclear Translocation to Facilitate p16 Expression

**Figure 6:**
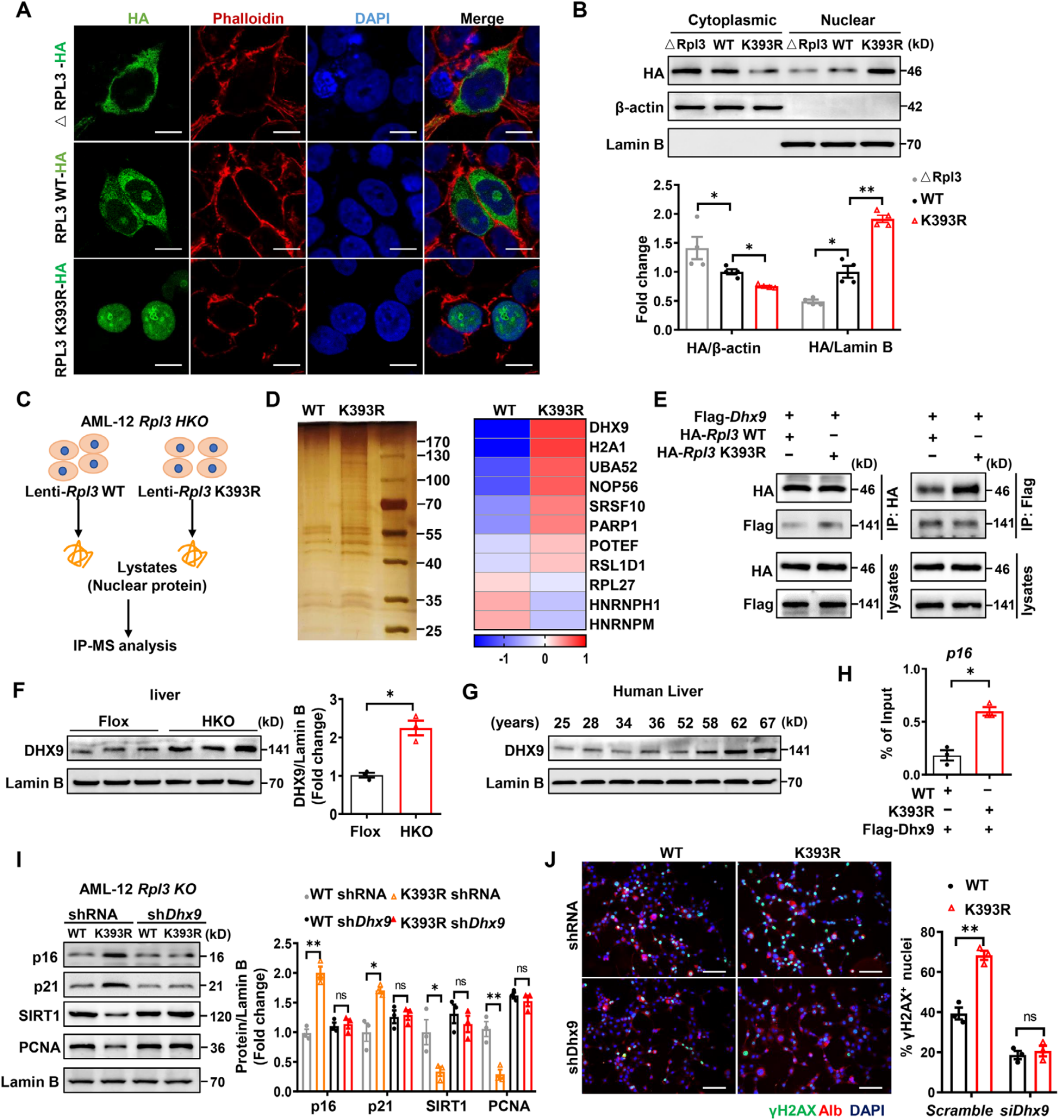
Removing SUMOylation of RPL3 promotes its nuclear translocation and enhances its interaction with DHX9. A) Representative image for immunostaining of RPL3 in HA‐*Rpl3* WT or K393R or △RPL3 transfected cells. Scale bars, 20 µm. B) *Rpl3* CRISPR KO AML12 cells were transduced with HA‐*Rpl3* WT or K393R or △RPL3 adenovirus for 48h. After being treated with H_2_O_2_ for 6 h, the cytosol and nuclear fractions were separated and subjected to western blot analysis with anti‐HA antibody (*n* = 4). C) A brief schematic diagram of identifying the downstream targets of nuclear RPL3. The equal nuclear fractions of *Rpl3* WT or K393R overexpressed cells from were incubated with anti‐HA overnight. The immunoprecipitates were harvested for quantitative IP‐MS. D) Silver staining of IP products from nuclear fractions of cells expressing HA‐*Rpl3* WT versus HA‐*Rpl3* K393R. The heat maps were generated based on enrichment of candidates in HA‐*Rpl3* WT versus HA‐*Rpl3* K393R. E) Representative co‐IP analysis evaluating the interaction between RPL3 and DHX9 in hepatocytes using exogenous Flag*‐Dhx9* and HA*‐Rpl3* WT or HA*‐Rpl3* K393R. F) Representative Western blot analysis for protein expression of DHX9 in liver homogenates from 12‐month‐old HKO and control mice (*n* = 3). G) Representative western blot analysis of DHX9 in liver tissue from individuals with different degrees of aging. H) ChIP results for the analysis of DHX9 binding activity to the *p16* promoter in hepatocytes transduced with Flag*‐Dhx9* and HA‐*Rpl3* WT or HA‐*Rpl3* K393R (*n* = 3). I) *Rpl3* CRISPR KO AML12 cells were infected with *Dhx9* shRNA or the control shRNA lentivirus, followed by transducing HA‐*Rpl3* WT or HA‐*Rpl3* K393R adenoviruses, respectively. After being treated with H_2_O_2_ (100 µm) for 6 h, whole‐cell lysates were subjected to Western blot analysis, and protein expression of p16, p21, SIRT1, and PCNA was determined (*n* = 3). J) Representative images of immunofluorescence staining (γH2AX, green; Alb, red; DAPI, blue) of cultures from the same group as in (I). Scale bars, 50 µm. All data are represented as mean ± SEM. Unpaired Student’ s *t*‐test was applied to the analysis of the data in (F), and (H). One‐way ANOVA with Bonferroni post hoc analysis was used in (B) and (I,J). ^*^ *p* < 0.05, ^**^ *p* < 0.01.

Given that ribosome‐free ribosome proteins would accumulate either in the cytoplasm or nucleus in response to stressful insult,^[^ ^29^, ^30^ ^]^ we thus embarked on the impact of SUMOylation on RPL3 cellular localization. Our *in‐silico* bioinformatic analysis suggested that RPL3 possesses a nuclear localization signal (NLS) close to its SUMOylation site (Figure S6A, Supporting Information). We first validated that depletion of this NLS (amino acids 391–397, △RPL3‐HA) completely abolished RLP3 nuclear translocation, and critically, disruption of its SUMOylation site (RPL3‐K393R) almost completely rendered RPL3 localized in the nucleus (**Figure** **6**A). Indeed, Western blot analysis of lysates from those cells confirmed the above observations (Figure 6B). Together, those findings suggest that SUMOylation represses RPL3 nuclear translocation. Since ribosomal RPL3 may play a role in protein synthesis, we thus examined two key regulators, eIF2a and 4E‐BP1, for translation initiation in HKO mice. Unexpectedly, hepatocyte senescence in HKO mice was independent of defective translation, as evidenced by the comparative levels of hepatic phosphorylated eIF2a or 4E‐BP1 between the HKO and control mice (Figure S6B, Supporting Information).

To identify the detailed mechanism by which RPL3 SUMOylation orchestrates hepatocyte senescence, we extracted nuclear proteins from *Rpl3* WT or K393R adenovirus transduced AML12 *Rpl3* KO cells after H_2_O_2_ treatment, and then subjected them to IP‐MS analysis as described (Figure 6C). Proteins with a 1.5‐fold abundance higher in the *Rpl3* K393R transduced group were considered as candidate cofactors. Specifically, several helicases, splicing factors, and ribosome proteins were identified, in which DHX9 ranked the highest one (Figure 6D). We thus selected DHX9 as a downstream target to address the impact of RPL3 SUMOylation on hepatocyte senescence.

We first checked the direct binding activity of RPL3 WT or K393R with DHX9. AML12 cells were transduced with an adenovirus expressing HA‐tagged *Rpl3* WT or K393R, along with Flag‐tagged DHX9, respectively. As expected, we identified an interaction between RPL3 and DHX9 (Figure S6C, Supporting Information). Notably, RPL3 K393R manifested higher efficiency to be co‐immunoprecipitated with DHX9 as compared to that of RPL3 WT (Figure 6E). In fact, the expression of DHX9 was increased in the liver from 12‐month‐old HKO mice as compared to the control mice (Figure 6F). Intriguingly, an age‐dependent up‐regulation of DHX9 expression was also noted in the livers from human subjects (Figure 6G).

DHX9 is a member of the DExH‐box helicase family, it also plays an active role in gene transcription by bridging transcriptional factors or cofactors and RNA polymerase II (RNA Pol II) or directly binding to the promoters of target genes.^[^ ^31^, ^32^ ^]^ It was noted that DHX9 strongly interacts with RNA Pol II in the presence of *Rpl3* K393R (Figure S6D, Supporting Information). Moreover, chromatin immunoprecipitation (ChIP) confirmed that DHX9 exhibited binding activity to the *p16* promoter (Figure S6E, Supporting Information), and overexpression of DHX9 in hepatocytes enhanced p16 expression following H_2_O_2_ insult (Figure S6F, Supporting Information). In contrast to *Rpl3*‐WT transduced cells, *Rpl3*‐K393R transduced cells displayed a significantly higher binding affinity of DHX9 to the *p16* promoter (Figure 6H), while knockdown of DHX9 (Figure S6G, Supporting Information) markedly abolished the difference in terms of the expression of senescent markers (Figure 6I) and DNA damage marker γH2AX (Figure 6J), between *Rpl3*‐WT and ‐K393R transduced cells. Overall, these data support that loss of RPL3 SUMOylation facilitates its nuclear translocation, where it binds to DHX9 to transcribe the expression of senescent markers such as p16.

### 2.7 Ectopic Ubc9 Expression Attenuates Hepatic Senescence and Inflammation

**Figure 7:**
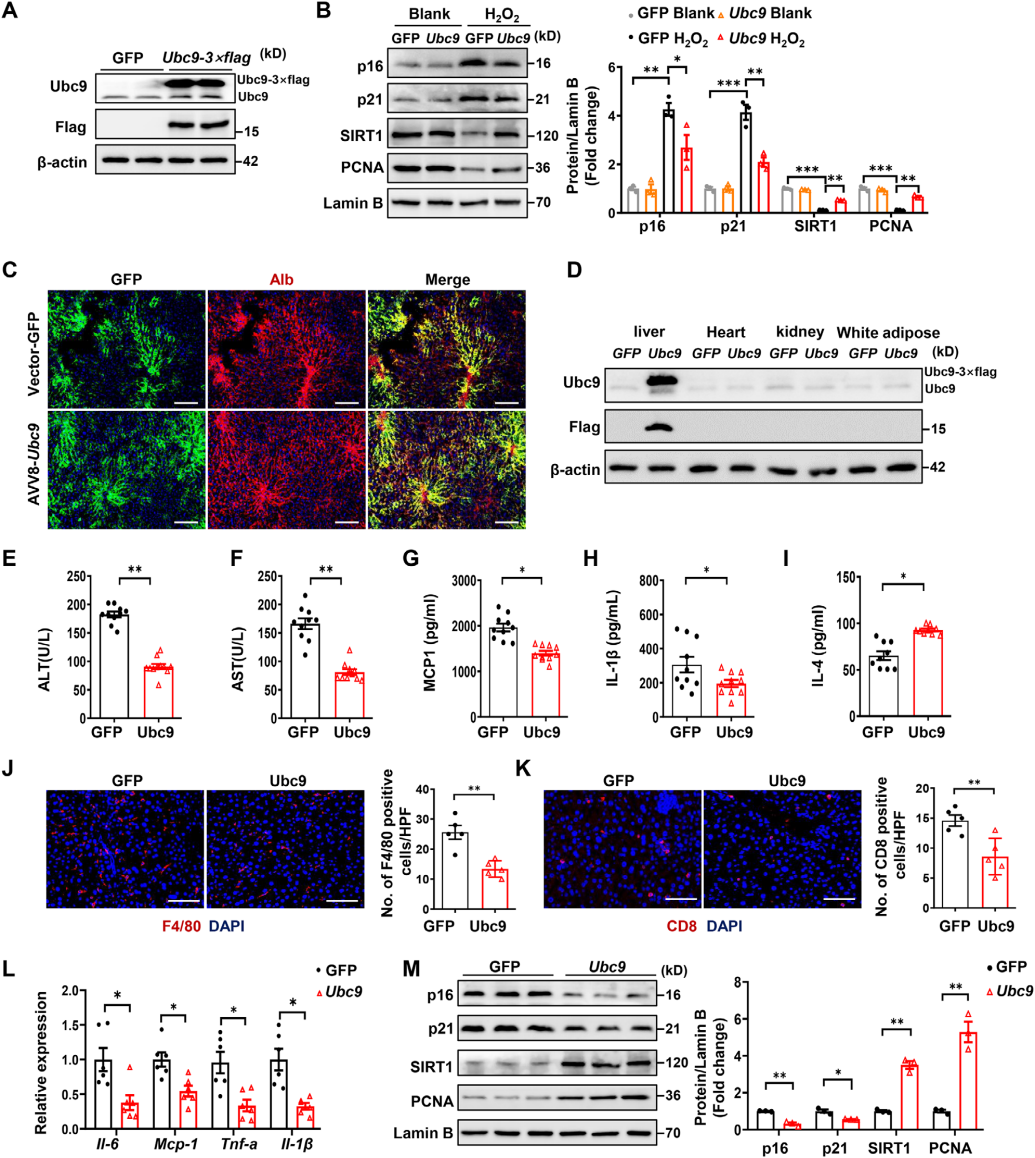
*Ubc9* overexpression by adeno‐associated virus attenuates hepatic senescence and inflammation. A) Representative western blot image showing the expression of Flag‐Ubc9 proteins in mouse primary hepatocytes infected with AdGFP and Ad3xFlag*‐Ubc9* for 48 h. B) Mouse primary hepatocytes were transduced with AdGFP and AdFlag*‐Ubc9* adenovirus for 48h. After being treated with H_2_O_2_ for 6 h, Cell lysates were subjected to western blot analysis, and protein expression of p16, P21, SIRT1, and PCNA was determined. C) Photomicrographs of frozen liver sections from mice after AAV8‐TBG‐GFP and AAV8‐TBG‐Flag*‐Ubc9* dosing. Scale bars, 100 µm. D) Representative western blot analysis of Flag‐Ubc9 protein expression in various organs of the mice in the indicated groups. E–I) Analysis of the serum levels of ALT (E), AST (F), MCP1(G), IL‐1β (H), and IL‐4 (I) in mice injected with AAV‐GFP or AAV‐Flag*‐Ubc9* (*n* = 9–10). J, K) Representative immunofluorescence staining images of F4/80 (J) and CD8 (K) of the liver sections of the indicated groups of mice (*n* = 5). Scale bars, 50 µm. L) Relative mRNA levels of inflammation response‐related genes in the liver of the mice in the indicated groups (*n* = 6). M) Western blot analysis of p16, p21, SIRT1, and PCNA in liver samples of the indicated groups of mice (*n* = 3). All data are represented as mean ± SEM. One‐way ANOVA with Bonferroni post hoc analysis was used in (B). Unpaired Student’ s *t*‐test was applied to the analysis of the data in (E–M). ^*^ *p* < 0.05, ^**^ *p* < 0.01, ^***^ *p* < 0.001.

Finally, we sought to explore the feasibility of ectopic Ubc9 expression against hepatocyte senescence. We first conducted Western blot analysis to validate the impact of ectopic Ubc9 expression on H_2_O_2_‐induced hepatocyte senescence. Primary hepatocytes were isolated from 2‐month‐old mice and then transduced with adenoviral particles carrying *Ubc9‐3xflag* (**Figures** **7**A, S7A, Supporting Information). As expected, ectopic Ubc9 significantly attenuated H_2_O_2_–induced expression of senescent markers (Figure 7B). Next, AAV8 carrying Ubc9 (AAV‐*Ubc9*) was injected into 18‐month‐old mice via the tail vein (Figure S7B, Supporting Information). Immunostaining (Figure 7C) and Western blotting (Figure 7D) confirmed ectopic Ubc9 expression in the livers after 4‐month of viral injection. Notably, the AAV‐*Ubc9* injected mice exhibited significantly decreased serum ALT, AST (Figure 7E,F), MCP‐1 and IL‐1β, but increased serum IL‐4 (Figure 7G–I) along with repressed macrophage and CD8 T cell (Figure 7J,K) infiltrations and attenuated expression of pro‐inflammatory factors (Figure 7L). All of which led to the attenuated hepatocyte senescence (Figure 7M) and liver injury (Figure S7C, Supporting Information). In summary, these findings support that Ubc9‐mediated SUMOylation could be a vital mechanism against hepatocyte senescence in clinical settings.

## 3 Discussion

Senescent hepatocytes undergo characteristic changes, including morphological alterations and metabolic reprogramming, driven by the epigenetic, transcriptional, and post‐transcriptional mechanisms.^[^ ^33^, ^34^ ^]^ Excessive high‐fat nutrition speeds up the aging process by intensifying inflammatory responses and boosting oxidative stress.^[^ ^35^, ^36^ ^]^ In general, the characteristics of enhanced p16 expression (p16^High^) predominantly occur in liver sinusoidal endothelial cells during the age‐related physiological aging process.^[^ ^37^, ^38^ ^]^ We found that environmental insults (e.g., H_2_O_2_ challenge) induce ectopic high levels of p16 expression in hepatocytes, accelerating hepatocyte senescence and causing lipid metabolic disorders. Our findings revealed that liver‐specific deletion of *Ubc9* accelerates hepatocyte aging and impairs liver function even under physiological conditions. Critically, upon the insult of an MCD diet, mice were characterized by exacerbated fatty liver disease, liver fibrosis, and hepatocyte senescence.

Previous studies indicated that environmental exposures and lifestyles are coupled with the changes of posttranslational modifications during the course of the lifespan.^[^ ^39^, ^40^, ^41^ ^]^ SUMOylation is an essential post‐translational regulatory mechanism strongly correlated to daily life processes.^[^ ^42^ ^]^ In particular, Ubc9‐mediated SUMOylation of CYP2E1 at lysine 410, plays a pivotal role in regulating CYP2E1 activity in alcoholic liver disease.^[^ ^43^ ^]^ Ubc9 also exhibits a remarkable versatility, capable of stabilizing Iκbα to inhibit NF‐κB signaling to modulate hepatic proinflammatory response induced by LPS.^[^ ^44^ ^]^ However, the intricate interplay between SUMOylation and the liver aging process is yet to be clearly elucidated. To identify the crucial SUMOylated substrates relevant to hepatocyte senescence, we conducted an integrative multiomic analysis by comparing the young and aged hepatocytes. Our studies demonstrated experimental evidence that hepatocyte aging is associated with changes in SUMOylated substrates and/or SUMOylation levels. Particularly, a marked decrease in SUMOylation levels in numerous ribosomal proteins, such as RPL3, in response to the induction of hepatocyte senescence was noted. Generally, RPL3 is involved in the formation of the large subunit of the ribosome and helps in the binding of transfer RNA (tRNA) to the ribosome during translation.^[^ ^45^ ^]^ Therefore, it plays a significant role in ensuring the accurate and efficient synthesis of proteins within cells.^[^ ^46^ ^]^

The assembly and maturation of ribosomes, primarily occurring within the nucleoli, are a highly coordinated process.^[^ ^47^ ^]^ During which the subunits are exported to the cytoplasm, where they undergo a series of protein additions and releases, to ultimately form the functional subunits.^[^ ^48^ ^]^ Ribosomal proteins exert their influence in cellular aging through two main mechanisms. First, they have the potential to disrupt the normal function of ribosomes, leading to abnormalities in protein synthesis,^[^ ^49^ ^]^ which in turn triggers stressful responses contributing to the aging process. Second, ribosomal proteins engage in interactions with key proteins associated with aging, such as MDM2, CDK4, and p53, thereby modulating their functions and directly impacting the cellular aging status.^[^ ^50^, ^51^ ^]^ In senescent human mesenchymal progenitor cells, nucleolar accumulation of RPL22 initiates heterochromatin decompaction and degradation of key proteins HP1γ and KAP1, triggering RPL22‐dependent rRNA transcription and driving cellular senescence.^[^ ^52^ ^]^ Furthermore, ribosomal proteins employ post‐transcriptional regulatory strategies to regulate cellular translation and function. For instance, the human enzyme METTL18 introduces a 3‐methylhistidine modification at position His245 of RPL3 to slow ribosomal movement specifically at Tyr codons, thereby ensuring the proper folding of newly synthesized proteins.^[^ ^53^ ^]^ It is worthy of note that disruption of ribosome biogenesis in the nucleoli, either induced by drugs or by metabolic stress, would result in the release of ribosome‐free RPL3.^[^ ^54^ ^]^ The ribosome‐free RPL3 accumulates in the nucleus, where it assumes the role of a transcription factors.^[^ ^55^ ^]^ In colorectal cancer, DUOX2 affects cell invasion and migration via interaction with RPL3, potentially by regulating the PI3K‐AKT pathway.^[^ ^55^ ^]^ These collective insights emphasize the multifaceted roles of ribosomal proteins and RPL3 in various cellular processes.

It is noteworthy that *Ubc9* deficiency resulted in elevated RPL3 expression in hepatocytes, which was likely directly induced by the aging process rather than by the enhancement of its stability through SUMOylation. Indeed, disruption of its SUMOylation motif at K393 did not result in a perceptible change in terms of its ubiquitylation. We also demonstrated experimental evidence that RPL3 possesses a nuclear localization signal (NLS), and disruption of this NLS abolished its nuclear localization. Interestingly, its SUMOylation site (K393) by chance is located within this nuclear signaling sequence. Therefore, we postulated that the covalent attachment of SUMO to its K393 (SUMOylation) may induce steric hindrance, thereby impeding its efficient nuclear translocation. Indeed, disruption of its SUMOylation motif by mutating K393 to arginine (K393R) significantly enhanced RPL3 nuclear translocation. We further found that ribosome‐free RPL3 accumulates in the nucleus, where it potently interacts with DHX9 to facilitate the transcription of senescent genes. Specifically, DHX9 acts as a DExD/H‐box helicase to maintain genomic stability by resolving RNA/DNA hybrids and R‐loops.^[^ ^31^ ^]^ Moreover, DHX9 can influence transcriptional activation by binding to the promoter regions of target genes.^[^ ^56^ ^]^ Intriguingly, we found that RPL3 forms a complex with DHX9, which then facilitates the recruitment of RNA Pol II, thereby culminating in the transcription of senescent genes such as p16.

To address the clinical relevance of our findings, AAV8 carrying Ubc9 (AAV‐*Ubc9*) was administered through tail vein injection into 18‐month‐old mice, and the mice were sacrificed after 4‐month of viral transduction. Excitingly, enhanced SUMOylation function by introducing ectopic Ubc9 expression markedly attenuated the hepatic aging process, as evidenced by the repressed expression of senescent markers, along with improved liver function and inhibited inflammatory responses. Although those results are encouraging, some limitations need to be further addressed in future studies. For example, our data revealed that SUMOylation does not affect ubiquitin‐mediated degradation of RPL3, but an enhanced RPL3 transcription was noted in *Ubc9*‐deficient hepatocytes. This unexpected observation suggests that SUMOylation may indirectly regulate RPL3 expression also at the transcriptional level during the aging process (Figure S7D, Supporting Information), but the detailed mechanism remains to be elucidated. Additionally, the exact E3 ligase that specifically facilitates RPL3 SUMOylation in hepatocytes is currently unknown, which also warrants further investigations. Knockout of Dhx9 can reverse the impact of RPL3 SUMOylation on the expression of hepatocyte senescence markers p16 and p21. Nevertheless, it remains uncertain whether Dhx9 can directly regulate p21 or other senescence genes, thereby influencing hepatocyte senescence and lipid metabolic disorders.

In summary, we demonstrated evidence supporting that the hepatocyte aging process is featured by the decreased Ubc9 expression along with an escalated decrease of protein SUMOylation. Therefore, mice with hepatocyte‐specific Ubc9 deficiency manifested exacerbated senescent phenotype and were more susceptible to MCD‐induced MASLD and steatohepatitis. Our mechanistic studies revealed that Ubc9 mediates RPL3 SUMOylation to attenuate its nuclear translocation, thereby repressing the transcription of senescent genes such as p16. These findings highlight that Ubc9‐mediated SUMOylation of RPL3 could be an unappreciated mechanism against hepatic aging and MASLD in clinical settings.

## 4 Experimental Section

### Mouse Model

The *Ubc9^fl/fl^* mice were generated as described previously.^[^ ^39^, ^40^ ^]^ For the generation of hepatocyte‐specific Ubc9 knockout (HKO) mice, the *Ubc9^fl/fl^* (Flox) mice were crossed with Alb‐Cre mice purchased from Jackson's Laboratory. Mice were housed in a SPF facility at the Tongji Hospital Research Building on a 12‐h light/12‐h dark cycle with ad libitum access to food and water. All animal studies were approved by the Tongji Hospital Animal Care and Use Committee.

### Human Liver Samples

Human liver tissues were collected at the Tongji Hospital of Tongji Medical College, Huazhong University of Science and Technology. The normal liver tissues were collected from individuals during the exploratory surgeries to exclude cancer or inflammatory diseases, or excessive alcohol consumption. Hepatic biopsy samples from MASLD patients were obtained during their histological examinations. Informed consent was obtained from each participant, and the studies were approved by the Institutional Review Board (IRB) at Tongji Hospital of Tongji Medical College, Huazhong University of Science and Technology (TJ‐IRB20210965).

### Primary Hepatocytes

Primary hepatocytes were isolated from 8‐week‐old male mice by a two‐step collagenase perfusion method as previously described.^[^ ^57^ ^]^ In brief, the mice were anesthetized and perfused with warm (37 °C) HBSS without calcium or magnesium ions, and then HBSS buffer supplemented with 0.5 mg mL^−1^ collagenase IV, calcium and magnesium ions via the portal vein. After digestion, the liver was carefully excised, and hepatocytes were softly released by filtering through a 100 µm cell strainer. The cells were next sedimented at 50 g for 2 min and washed two times with DMEM/F12. After resuspended with DMEM/F12 containing 10% FBS and 1% penicillin‐streptomycin, the hepatocytes were gently seeded onto plates coated with collagen and were cultured in a 5% CO2 incubator. All experiments in primary hepatocytes were performed within 24 h of plating the cells.

### Cell Culture

AML12 (catalog SCSP‐550) and HEK293T (catalog SCSP‐502) cells were obtained from the Cell Bank of the Chinese Academy of Sciences (Shanghai, China). The AML12 cells were cultured in DMEM/F12 medium supplemented with 10% fetal bovine serum (FBS), 1% penicillin–streptomycin, 1 mg mL^−1^ insulin, 0.55 mg mL^−1^ transferrin, 0.5 ng mL^−1^ selenium, and 40 ng mL^−1^ dexamethasone. To confirm their hepatocyte identity, these AML12 cells were further subjected to immunofluorescence staining using an antibody against the hepatocyte‐specific marker HNF4α. HEK293T cells were cultured in DMEM with 10% FBS and 1% penicillin‐streptomycin. All cells were cultured at 37 °C in a humidified incubator with 5% CO2. To establish a hepatic senescent model in vitro, mouse primary hepatocytes and AML12 cells were stimulated with hydrogen peroxide (H_2_O_2_,100 or 200 mm) 6 h.

### Adeno‐Associated Virus 8‐Mediated Ectopic Expression of RPL3 WT or Rpl3 K393R in Mice

For exogenous RPL3 WT or RPL3 K393R expression, the corresponding mouse RPL3 sequence with a C‐terminal HA tag was cloned into an AAV8 vector under the control of a mouse albumin promoter to generate AAV8‐TBG‐RPL3 WT or RPL3 K393R, respectively. The adenovirus was administered by tail vein injection into 18‐week‐old C57BL/6J male mice at a titer of 1 × 10^9^ IFU per mouse. The senescence phenotype was analyzed after 4‐month of viral transduction.

### Lentiviral Packaging and Transducing (lentiCRISPR v2 system) in AML12 Cells to Establish an RPL3 Knockout Stable Cell Line

The following sequence 5‐CGT AAG GTG AGA CTG ATC CTG GG‐3 of the mouse RPL3 gene was selected as the target. Oligos were amplified and ligated into the lentiCRISPRv2 plasmid. Plasmids for lentiCRISPR v2, psPAX2, and pMD.2G were co‐transfected into HEK293T cells at a ratio of 4:3:1 for 48 h, and the culture media were collected. Viral particles within the supernatants were purified and then added into the culture medium with polybrene (8 mg mL^−1^) to transduce AML12 cells for 24 h. Puromycin (2 mg mL^−1^) was used for sorting and selecting RPL3 knockout cells.

### Lentiviral Packaging and Transducing (pLKO.1‐TRC lentiviral shRNA System)

The pLKO.1‐TRC lentiviral shRNA system (Addgene, pLKO.1‐TRC.mKO2, Plasmid #85208) was used for DHX9 knockdown. shRNA targeting sequence for mouse DHX9:

(#1: GCCGTTCTCGTGGAAGGTTGG, #2: GCTGCCAGAGACTTTGTTAAC) were cloned into the pLKO.1 plasmid. pLKO.1, pAX.2, and pMD.2G were co‐transfected into HEK293T cells at a ratio of 4:3:1 for 48 h. The viral supernatant was collected, purified, and used to transduce AML12 cells in the presence of polybrene (8 µg mL^−1^) for 24 h. Western blot analysis was used to assess knockdown efficiency, and a short hairpin RNA (shRNA) was used as a negative control.

### Administration of Senolytics into HKO Mice

Dasatinib (5 mg kg^−1^, drug/body weight) and Quercetin Q (50 mg kg^−1^) (DQ) were dissolved in 100 µL10% PEG400. The prepared dasatinib and quercetin Q were administered to 18‐month‐old HKO mice by oral gavage every 2 weeks for a period of 2 months. HKO mice administered with the control vehicle served as controls. The mice were next sacrificed for comparative analysis of senescent parameters.

### Senescence‐Associated β‐galactosidase Staining

The cells were treated with H_2_O_2_ (100 µm) for 6 h. After washing with PBS, the cells were fixed in 4% paraformaldehyde for 10 min, followed by incubating with X‐gal mixture at 37 °C for 24 h according to the manufacturer's protocol. The frequency of positive cells was determined according to the instructions from the provider.

### Preparation of Cytoplasmic and Nuclear Fractions

The prepared cells were rinsed with cold PBS and then harvested. The harvested cells were resuspended in 5 volumes of cold Buffer A (10 mM KCl, 0.1 mM EDTA (pH 7.9), 0.1 mM EGTA, 10 mM HEPES, 1 mM DTT, 0.15% NP‐40, and protease inhibitor cocktail, and were lysed on ice for 10 min. The homogenates were centrifuged for 2 min at 12 000 g, and cytoplasm fraction was collected in the supernatants. Next, Buffer B containing 400 mm NaCl, 10 mM KCl, 1.5 mM MgCl2,10 mM Tris pH 7.9, 0.4% Triton X‐100, and protease inhibitor cocktail was added to the homogenized nuclear pellets at the bottom and solubilized sufficiently on ice for 30 min (with a vigorous vortex every 10 min). The homogenates were centrifuged at 12 000 g at 4 °C for 15 min, and the nuclear extracts were collected from the supernatants.

### RNA‐seq and Data Processing

To investigate gene expression differences, total RNA was first isolated from liver tissue or hepatocytes using the TRIzol method and its quality assessed. Subsequently, cDNA libraries were then constructed for single‐end sequencing on the BGISEQ 500 platform. The sequencing reads were mapped to Ensembl mouse reference genomes via HISAT2. Gene expression was quantified as FPKM values via FeatureCounts (http://subread.sourceforge.net/). Differential gene expression analysis was conducted with DESeq2, identifying differentially expressed genes (DEGs) based on a fold‐change threshold >1.5 and an adjusted *p*‐value <0.05. Gene Ontology (GO) functional enrichment and Kyoto Encyclopedia of Genes and Genomes (KEGG) pathway enrichment analyses were performed using the clusterProfiler R package. Gene Set Enrichment Analysis (GSEA, v4.3.2) was conducted using default parameters, including 1,000 permutations for significance testing of gene sets and the Signal2Noise metric for gene ranking.

### Mass Spectrometry Analysis

Nuclear proteins were extracted from HA‐Rpl3 WT or HA‐Rpl3 K393R transduced AML12 cells following H_2_O_2_ treatment. Equal amounts of nuclear proteins were subjected to co‐immunoprecipitation using a polyclonal anti‐HA antibody, and the precipitates were applied to liquid chromatography–tandem mass spectrometry (LC‐MS/MS) analysis. The interacting targets should fulfill the condition that the candidates should be presented in the HA‐Rpl3 WT but increased in the HA‐Rpl3 K393R transduced cells.

### Proteomic Analysis of SUMOylated Substrates

For identification of SUMO1‐conjugated proteins, primary hepatocytes isolated from young (2 months) or aged (22 months) mice were lysed with IP lysis buffer. Co‐IP was carried out using a polyclonal anit‐SUMO1 antibody, respectively. The same amount of resulting precipitates was subjected to LC‐MS/MS analysis to identify SUMO1‐cojugated substrates and comparative analysis of the changes of SUMOylation substrates and levels. For screening Ubc9‐bound proteins, primary hepatocytes isolated from young (2 months) mice were transduced with Flag‐Ubc9 or vector‐Flag adenoviruses at a multiplicity of infection (MOI) of 50. After 48 h of transduction, the cells were subjected to preparation of cell lysates in lysis buffer, followed by Co‐IP using a polyclonal anti‐Flag antibody. The resulting co‐IP products were next analyzed by LC‐MS/MS to identify Ubc9‐bound proteins. SUMOylated peptides were considered differentially expressed if they met one of the following criteria: (1) fold change > 1.5 with a *p* value < 0.05, or (2) detected only in one group. GO functional enrichment analysis was conducted using Goatools (https://github.com/tanghaibao/Goatools).

### Western Blot Analysis

Total proteins from tissues and cells were prepared using the RIPA lysis buffer (P0013E; Beyotime Biotechnology; Shanghai, China) according to the instructions. The prepared proteins were quantified using a BCA kit, and 2 mg of proteins from each sample were separated by SDS‐PAGE, and the separated proteins were transferred onto PVDF membranes as previously reported.^[^ ^58^ ^]^ The PVDF blots were blocked in 5% milk for 1 h at room temperature and incubated overnight at 4 °C with an indicated primary antibody, followed by incubating with a dye‐labeled secondary antibody for 1 h at room temperature. The blots were visualized using an enhanced chemiluminescence kit and recorded with an Image Lab imaging system. The antibodies used are listed in Table S1 (Supporting Information).

### Quantitative PCR Analysis

Total RNAs were extracted from cells or tissues using the Trizol reagent. A cDNA Synthesis Kit was employed to synthesize the corresponding cDNAs. Quantitative PCR assays were carried out using the SYBR Green PCR Master Mix with a Real‐Time PCR System (LightCycler 480 Instrument II, Roche; Basel, BS, Switzerland). Relative mRNA levels were normalized by β‐actin and determined by the formula 2‐△△CT as reported.^[^ ^59^ ^]^ The primer sequences used are listed in Table S2， (Supporting Information).

### Chromatin Immunoprecipitation (ChIP) Assay

AML12 cells were transduced with Flag‐Dhx9 and HA‐WT Rpl3 or HA‐Rpl3 K393R adenoviruses, followed by H_2_O_2_ treatment as described earlier for 1 h. After cross‐linked with 1% formaldehyde for 5 min at 37 °C, the cells were harvested in SDS Lysis buffer containing PMSF (Beyotime Biotechnology, Shanghai, China), and chromatin fragmentation was achieved by sonication at 4 °C. The nuclear extracts were immunoprecipitated with a polyclonal anti‐Flag antibody or a control IgG overnight at 4 °C with rotation. The mixtures were next incubated with Protein A+G Agarose beads for 2 h at 4 °C. After washing, the DNA‐protein complexes were eluted out with elution buffer. The eluted DNA segments were purified and used for PCR analysis of the indicated promoter regions. The primers used for ChIP‐PCR were listed in Table S 2(Supporting Information), and +1 defined as the transcriptional start site.

### Statistical Analysis

Statistical analysis was performed using GraphPad Prism 9, with data presented as mean ± SEM. For in vitro cellular experiments, three biologically independent replicates with consistent results were performed. For comparisons between two independent groups, a two‐tailed unpaired Student's *t*‐test was used. For multiple group comparisons, statistical significance was assessed using one‐way ANOVA. A *p* value < 0.05 was considered significant in all experiments, with ^*^ *p* < 0.05, ^**^ *p* < 0.01, and ^***^ *p* < 0.001 indicating levels of significance. The statistical parameters and corresponding *p* values were indicated in the figure legends.

## Conflict of Interest

The authors declare no conflict of interest.

## Author Contributions

H.X., Z.G., X.L. contributed equally to this work. H.X. and C.Y. designed the research. H.X., Z.G., and X.L. conducted experiments and wrote the manuscript. S.Z., Y.H., J.G., P.Q., L.Z., J.Z., T.X., H.T. and J.S. provided experimental support. Q.Y., S.Z., Y.L., P.Y., M.S.A. and Q.G. assisted in data analysis and discussion. J.D., H.Y. and S.L recruited subjects and collected samples. S.F. and C.W. edited the manuscript. C.W. supervised and funded the project.

## Supporting information



Supporting Information

## Data Availability

The data that support the findings of this study are available from the corresponding author upon reasonable request.
