# Supplementary material for: Ubc9‐Mediated SUMOylation of RPL3, an Unappreciated Mechanism against Hepatocyte Senescence by Repressing the DHX9‐p16 Axis
Source: Adv Sci (Weinh). 2025 Oct 20;13(1):e10240. doi: 10.1002/advs.202510240 (PMC12767081; doi:10.1002/advs.202510240)
Supplement: Supplementary file 1 — Supporting Information [file ADVS-13-e10240-s001.pdf]

Ubc9-mediated SUMOylation of RPL3, an unappreciated mechanism against hepatocyte senescence by repressing the DHX9-p16 axis

Hao Xie[1, 2, #], Zhichao Gao[1, #], Xin Liu[3, #], Shiyi Zhang[1], Yuhan Wang[1], Jia Gao[1], Ping Qi[1], Lu Zhang[1], Jiawei Zhao[1], Tian Xiong[4], Teng Huang[1, 4], Jia Song[1, 5], Qilin Yu[1], Shu Zhang[1], Yanjun Liu[6], Ping Yang[7], Maryam S. Al-Motawa[8], Quan Gong[9], Junfeng Dong[10], Hao Yin[10,*], Fei Sun[1,8,*], Shiwei Liu[7,*], Cong-Yi Wang[7,8,11,*]

[1]Department of Respiratory and Critical Care Medicine, the Center for Biomedical Research, NHC Key Laboratory of Respiratory Diseases, Tongji Hospital, Tongji Medical College, Huazhong University of Science and Technology, Wuhan, China

[2]Department of Clinical Laboratory, Institute of Translational Medicine, Renmin Hospital of Wuhan University, Wuhan, Hubei, 430060 China

[3]Department of Interventional Radiology, Renmin Hospital of Wuhan University, Wuhan, China.

[4]Department of Geriatrics, Tongji Hospital, Tongji Medical College, Huazhong University of Science and Technology, Wuhan, China.

[5]Reproductive Medicine Center, Tongji Hospital, Tongji Medical College, Huazhong University of Science and Technology, Wuhan, Hubei, China.

[6]The Center for Obesity and Metabolic Health, Affiliated Hospital of Southwest Jiaotong University, the Third People's Hospital of Chengdu, 82 Qinglong Road, Chengdu 610031, Sichuan China

[7]Shanxi Bethune Hospital, Shanxi Academy of Medical Science, Tongji Shanxi Hospital, Third Hospital of Shanxi Medical University, the Key Laboratory of Endocrine and Metabolic Diseases of Shanxi Province, Taiyuan, China.

[8]Diabetes Research Center, Qatar Biomedical Research Institute (QBRI), Hamad Bin Khalifa University (HBKU), Doha 34110, Qatar.

[9]Department of Immunology, School of Medicine, Yangtze University, Jingzhou, Hubei 434023, China.

[10]Organ Transplant Center, Shanghai Changzheng Hospital (Second Affiliated Hospital of Naval Medical University), Shanghai, China.

[11]The Center for Biomedical Research, Tongji Hospital Research Building, Tongji Hospital, Wuhan, China.

#These authors contributed equally to this work.

*Correspondence should be addressed to Drs. Cong-Yi Wang (wangcy@tjh.tjmu.edu.cn; cwang@hbku.edu.qa) and Fei Sun (phil_sunfei@tjh.tjmu.edu.cn), Shi-Wei Liu (liushiwei@sxbqeh.com.cn), and Hao Yin (yinhaoshanghai@163.com).

This PDF file includes:

Figs. S1 to S7

Tables S1 to S2

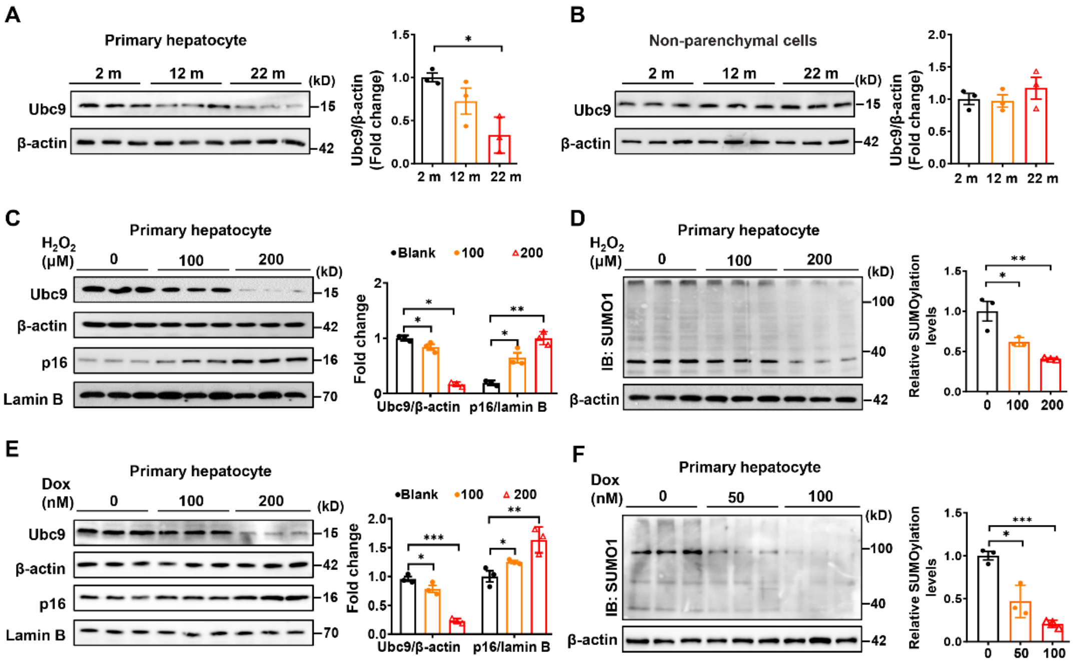

Figure S1. Generation of Hepatocyte specific *Ubc9* knockout mice. A, B) Representative western blot analysis of Ubc9 in primary hepatocytes (A) and non-parenchymal cells (B) isolated in liver tissue from 2-month, 12-month and 22-month-old mice. C, D) Primary hepatocytes isolated from 2-month normal mice were treated with H_2O_2 for the indicated concentrations. The protein levels of Ubc9, p16 (C) and SUMO1-conjuncted substrates (D) were determined by Western blot. E, F) Primary hepatocytes, isolated from 2-month-old normal mice, were subjected to treatment with Dox at the specified concentrations. Subsequently, the protein levels of Ubc9, p16 (E), and SUMO1-conjugated substrates (F) were assessed via Western blot analysis. All data are represented as mean ± SEM. The data in (A-F) were analyzed by One-way ANOVA with Bonferroni post hoc analysis. *$p < 0.05$, **$p < 0.01$, ***$p < 0.001$.

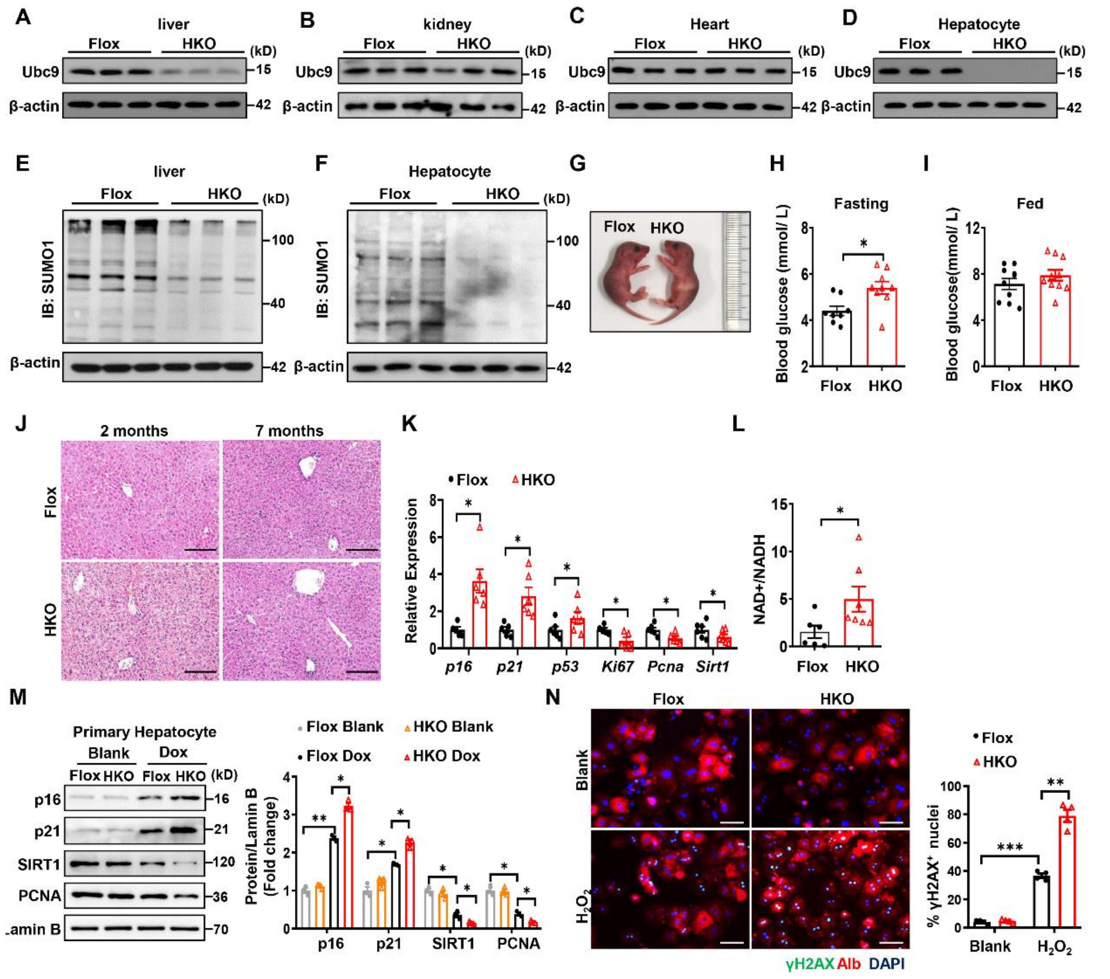

Figure S2. **Loss of Ubc9 increases hepatocyte senescence and impairs liver function.** A-D) Representative western blot analysis of Ubc9 protein expression in liver (A), kidney (B), heart (C) hepatocytes (D) isolated from the liver of Flox and HKO mice (*n* = 3). E, F) Representative western blot images of SUMOylated proteins in liver homogenates (E) and hepatocytes (F) isolated the liver of HKO and control mice. G) A morphologic photograph of newborns in the indicated groups. H, I) Fasting (H) and fed blood glucose (I) of 12-month-old HKO and control mice (*n* = 8-10). J) H&E staining of liver section from 2- and 7-month HKO and control mice. K) Relative mRNA levels of genes related to senescent and proliferative markers in the indicated groups (*n* =3). L) Intracellular NAD+/NADH levels in liver homogenates of 12-month-old HKO and

control mice (*n* = 6-7). M) Primary hepatocytes isolated from 2-month-old HKO or Flox mice were treated with doxorubicin (Dox,100 nM) for 24 h. Cell lysates were subjected to western blot analysis and protein expression of p16, p21, SIRT1 and PCNA was determined (*n* = 3). N) Representative images of immunofluorescence staining (γH2AX, green; Alb, red; DAPI, blue) of primary hepatocytes following 6-h treatment with H_2O_2 (*n* = 4). Quantification of γH2AX-positive nuclei per total nuclei from individual images (right panel). Scale bars, 20 μm. Unpaired Student' s t-test was used to analysis of the data in (H), (I), (K) and (L). All data are represented as mean ± SEM. The data in (M) and (N) were analyzed by One-way ANOVA with Bonferroni post hoc analysis. $^*p < 0.05$, $^{**}p < 0.01$.

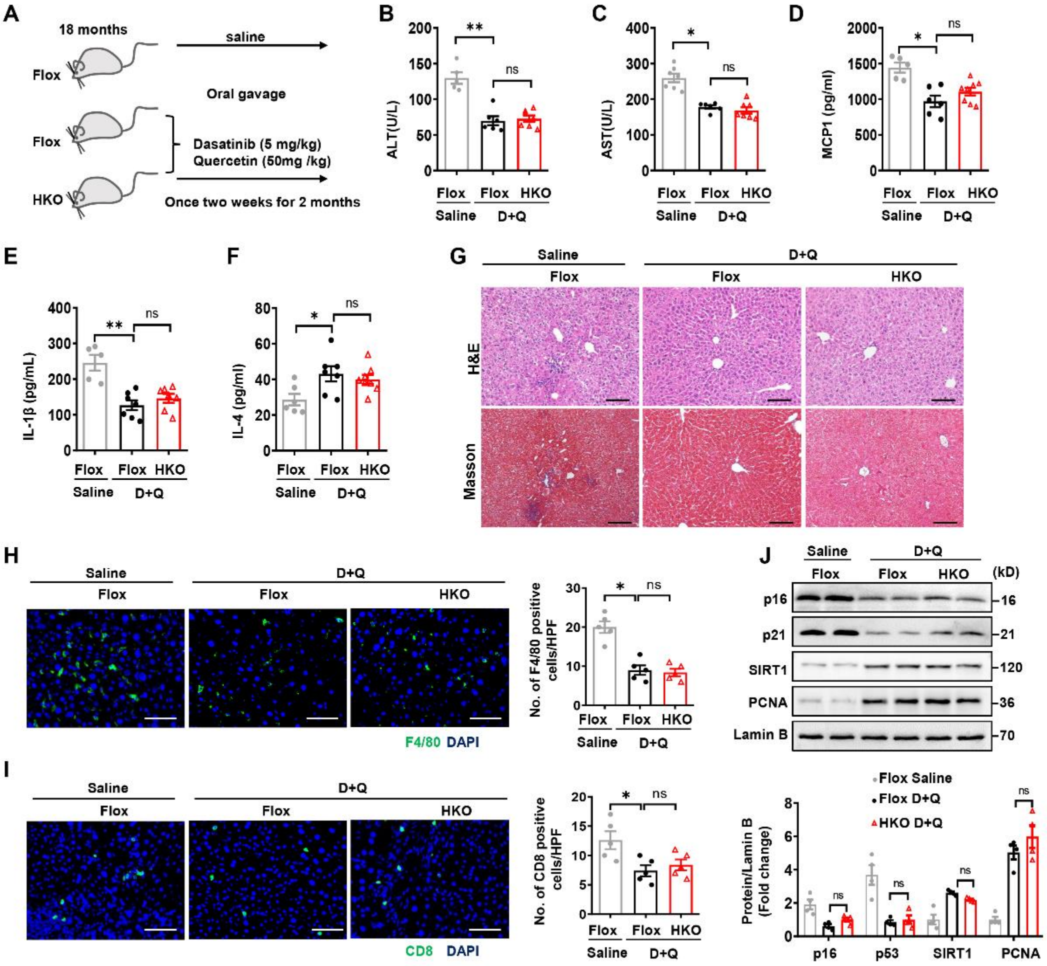

A
18 months
saline
Flox
Oral gavage
Flox
Dasatinib (5 mg/kg)
Quercetin (50mg /kg)
HKO
Once two weeks for 2 months
B
ALT(U/L)
C
AST(U/L)
D
MCP1 (pg/ml)
E
IL-1β (pg/mL)
F
IL-4 (pg/ml)
Flox
Flox
HKO
Saline
D+Q
ns
G
Saline
D+Q
Flox
Flox
HKO
H&E
Masson
H
Saline
D+Q
Flox
Flox
HKO
F4/80 DAPI
No. of F4/80 positive cells/HPF
I
CD8 DAPI
No. of CD8 positive cells/HPF
J
(kD)
p16
p21
SIRT1
PCNA
Lamin B
16
21
120
36
70
Flox Saline
Flox D+Q
HKO D+Q
Protein/Lamin B (Fold change)
p16
p53
SIRT1
PCNA

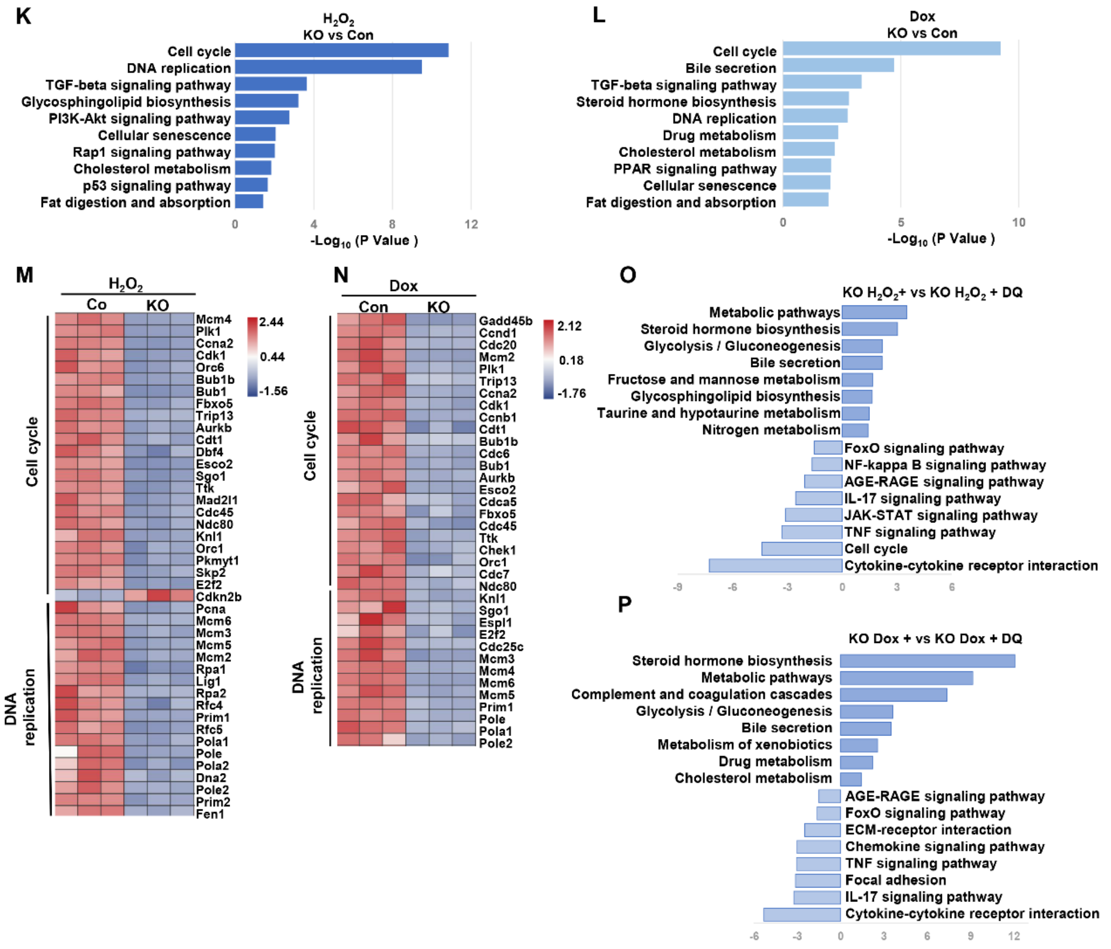

Figure S3. The impact of RPL3 SUMOylation on hepatocyte function relies on the modulation of hepatocyte senescence. (A) Diagram outlining the experimental design for evaluating the impact of *Ubc9* deletion on hepatocyte senescence.18-month-old HKO and control mice received the senolytic drugs dasatinib (5 mg/kg body mass) and quercetin (50 mg/kg body mass) once 2 weeks for 2 months via oral gavage. Control mice were treated with vehicle (10% polyethylene glycol 400 solution) only. (B-F) The serum levels of ALT (B), AST (C) and inflammatory factors (D-F) of the mice in the indicated group (n = 5-7). (G) Representative H&E staining and Masson staining of the liver sections of the mice in the indicated group. Circled regions showed mild perisinusoidal fibrosis. Scale bars, 100 μm. H, I) Representative immunofluorescence

staining images of F4/80 (H) and CD8 (I) of the liver sections of the mice in the indicated group. The right panels showed quantification of F4/80 and CD8 positive cells in each filed. Scale bars, 50 μm. (J) Representative western blot showed the protein levels of p16, p21, SIRT1 and PCNA in liver homogenates of the mice in the indicated group (*n* = 5-7). K, L) KEGG pathway enrichment analysis of mRNA sequencing data reveals differentially regulated pathways in primary hepatocytes from 2-month-old HKO and Flox mice following H_2O_2 or Dox treatment. M, N) RNA-Seq heatmap visualization analyzing differentially expressed genes in primary hepatocytes isolated from 2-month-old HKO and Flox mice following induction with H_2O_2 or Dox. O, P) KEGG pathway enrichment analysis of mRNA sequencing data revealed differentially regulated pathways in vehicle- and D+Q-treated primary hepatocytes from 2-month-old HKO, followed by H_2O_2 or Dox treatment. All data are represented as mean ± SEM. The data in (B-F) and (H-I) were analyzed by One-way ANOVA with Bonferroni post hoc analysis. *$p < 0.05$, **$p < 0.01$.

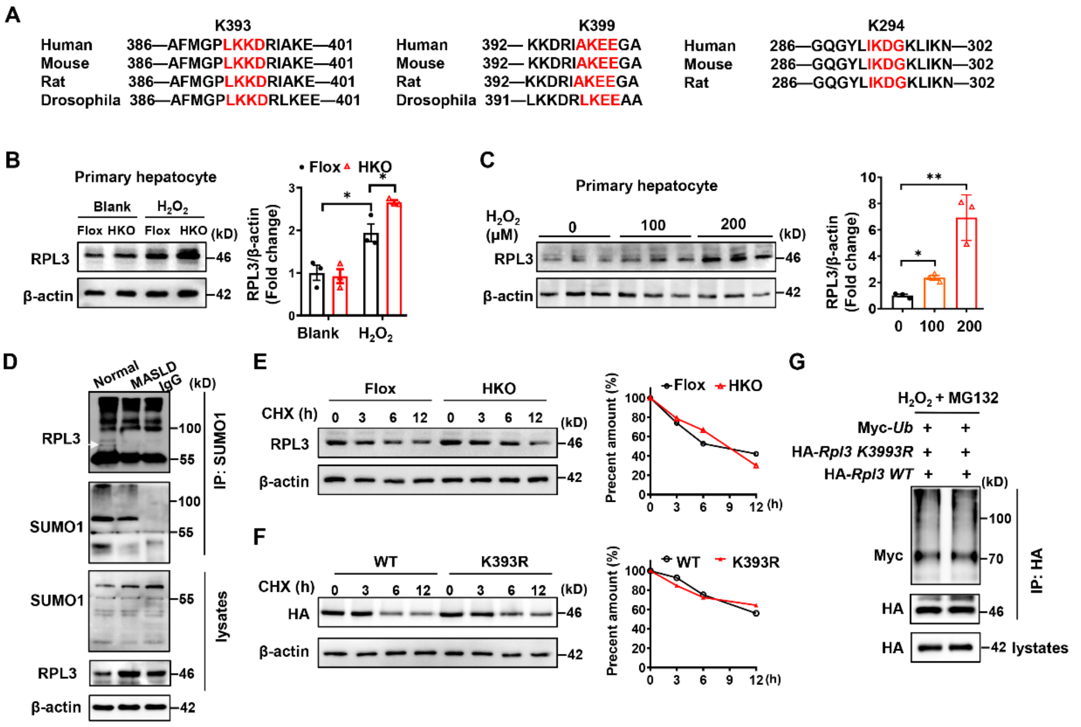

Figure S4. RPL3 is a novel SUMOylation target. A) Alignment of RPL3 amino acid sequences from several species with mouse RPL3 SUMOylation site. B) Primary hepatocytes isolated from 2-month-old HKO or Flox mice were treated with H_2O_2 (100 μM) for 6 h. Cell lysates were subjected to western blot analysis and protein expression of RPL3 was determined (n = 3). C) Representative Western blot analysis of RPL3 expression in primary hepatocytes treated with H_2O_2 for the indicated concentrations (n = 3). D) Co-IP assay revealed differential SUMOylated levels of RPL3 level in liver samples from patient with MASLD and control group. E) Primary hepatocytes isolated from 12-month-old HKO or Flox mice were exposed to cycloheximide (CHX, 100 μg/mL) for the indicated times and cell lysates were subjected to western blot for analysis of RPL3 expression. F) Primary hepatocytes transfected with indicated adenovirus were treated with CHX for different periods. Cell lysates were subjected to western blot for analysis of exogenous RPL3 expression. G) Ubiquitination levels of *Rpl3* WT or K393R

in hepatocytes treated with MG132 (10 μM). All data are represented as mean ± SEM. One-way ANOVA with Bonferroni post hoc analysis was used to analysis of the data in (B) and (C). *$p < 0.05$, **$p < 0.01$.

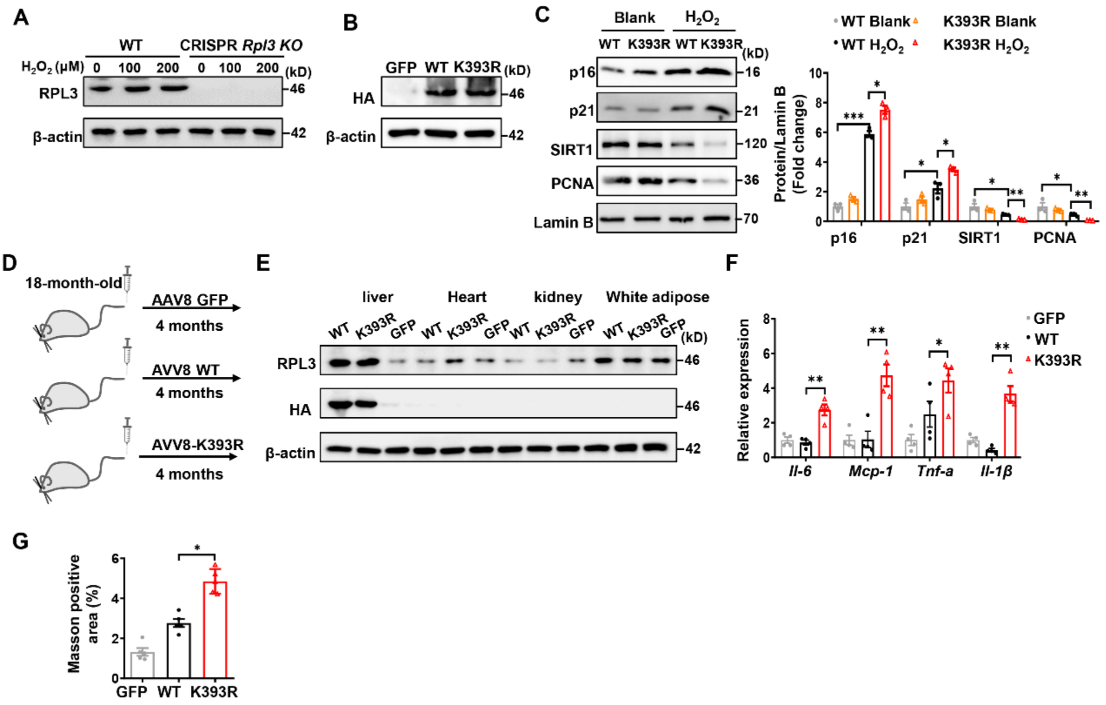

Figure S5. Disruption of RPL3 SUMOylation aggravates hepatocyte senescence.

A) WT and *Rpl3* CRISPR KO AML12 cells were treated with different concentrations of H_2O_2 (0, 100, and 200 μM) for 6 h. Protein levels of RPL3 were analyzed by immunoblotting. B) Representative western blot analysis of exogenous HA-*Rpl3* WT or HA-*Rpl3* K393R expression in *Rpl3* CRISPR KO AML-12 cells infected by RPL3 WT or Rpl3 K393R adenovirus. C) *Rpl3* CRISPR KO AML12 cells were infected by Rpl3 WT or K393R adenovirus and then treated for 6 h with H_2O_2 (100 μM). Protein levels of p16, p21, SIRT1 and PCNA were determined by western blot analysis. D) Experimental design for in vivo direct *Rpl3* WT or K393R delivery by tail vein virus injection. E) Representative western blot analysis of exogenous HA-*Rpl3* WT or HA-*Rpl3* K393R expression in tissue from mice injected with indicated virus. F) Relative mRNA levels of inflammation response related genes in liver tissue of the mice in the indicated groups (n = 4). G) Quantification of positive area for Masson staining

corresponding to Figure 5E (n = 5). All data are represented as mean ± SEM. The data in (C) (F) and (G) were analyzed by One-way ANOVA with Bonferroni post hoc analysis. *$p < 0.05$, **$p < 0.01$, ***$p < 0.001$.

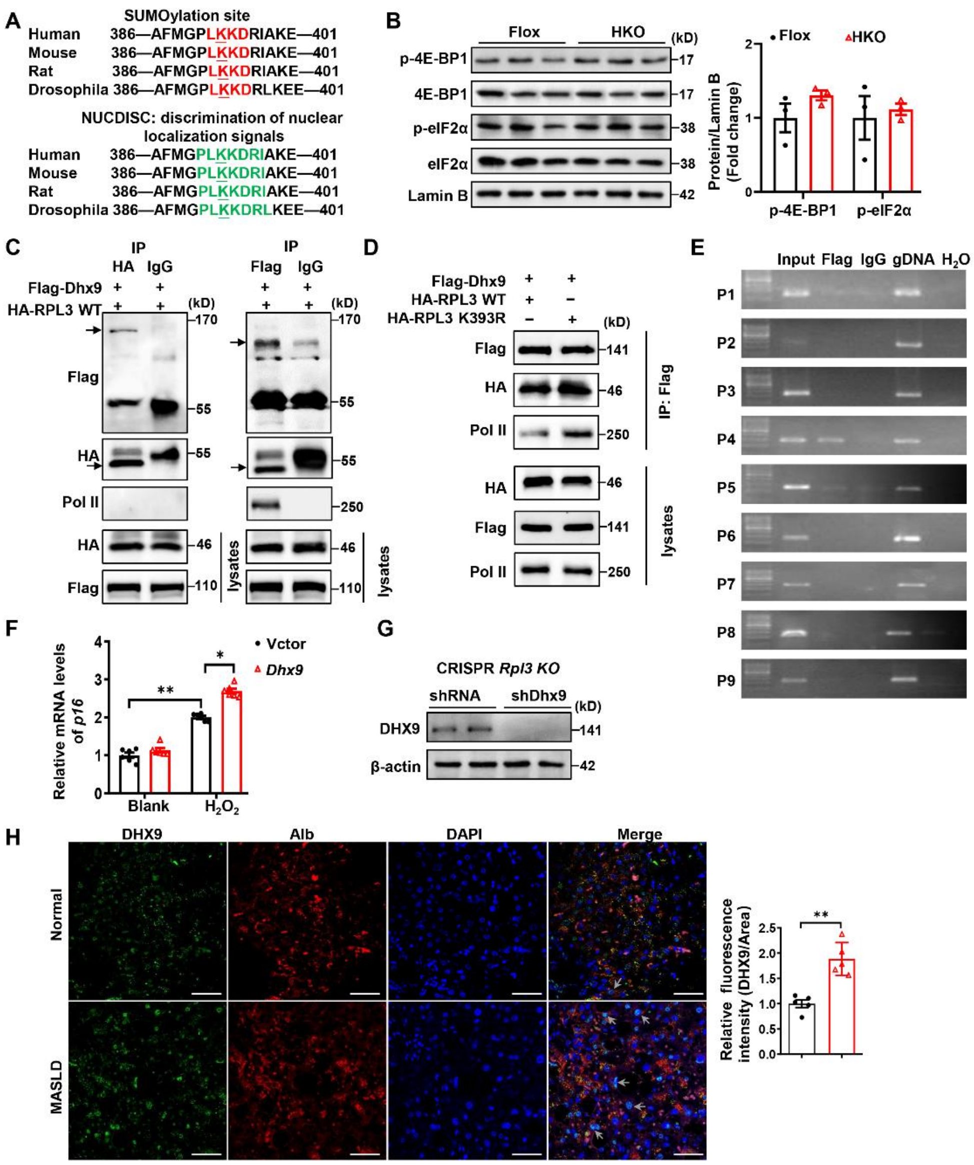

Figure S6. The disruption of RPL3 SUMOylation enhances its binding to Dhx9.

A) Alignment of RPL3 amino acid sequences from several species with mouse RPL3 SUMOylation site and predicted nuclear localization signals. B) Representative western blot analysis of 4E-BP1, phosphorylated 4E-BP1, eIF2α and phosphorylated eIF2α expression in liver of 12-month-old HKO and control mice (*n* = 3). C) Representative co-IP analysis evaluating the interaction between DHX9 and RPL3 or Pol II in hepatocytes using exogenous *Flag-DHX9* and *HA-Rpl3* WT, and blotted as indicated. D) Representative co-IP analysis evaluating the interaction between DHX9

and Pol II in hepatocytes stably expressing Flag-*Dhx9* and HA-*Rpl3* WT or HA-*Rpl3* K393R. E) ChIP results for the analysis of DHX9 binding activity to the p16 promoter. F) Relative mRNA levels of p16 in hepatocytes stably expressing Flag-*Dhx9* with or without H_2O_2 treatment (n = 6). G) Verification of the efficiency of shRNA against *Dhx9* at the protein level in hepatocytes. H) Representative images of DHX9 immunofluorescence staining in liver sections from patient with MASLD and control subjects. Scale bars, 100 μm. All data are represented as mean ± SEM. The data in (A) and (E) were analyzed by One-way ANOVA with Bonferroni post hoc analysis. *p < 0.05, **p < 0.01.

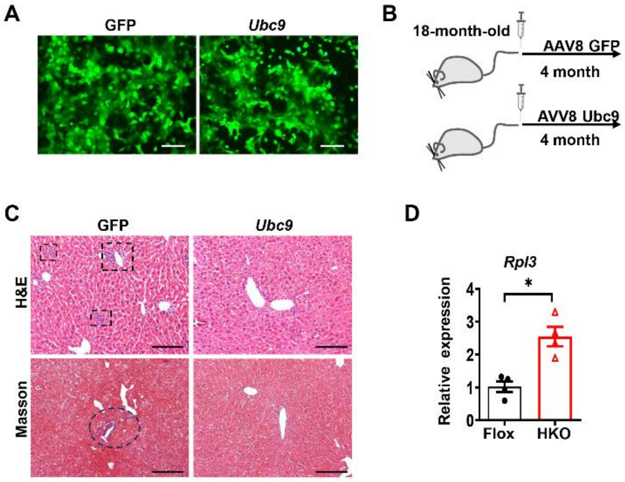

Figure S7. Ubc9 overexpression by adeno-associated virus attenuates hepatic senescence and inflammation. A) Photomicrographs of AML12 hepatocyte cell line infected with AdGFP or AdFlag-*Ubc9*. Scale bars, 50 μm B) Experimental design for in vivo direct GFP or *Ubc9* delivery by tail vein virus injection. C) Representative H&E and Masson staining of the liver sections of the mice in the indicated groups. The boxed area in the figure shows the inflammatory response. Circled regions showed prominent pericentral and perisinusoidal fibrosis Scale bars, 100 μm. D) Real-time PCR for analysis of *Rpl3* in liver tissue of 12-month-old HKO and control mice (n = 4). All data are represented as mean ± SEM. Unpaired Student' s t-test was used to analysis of the data in (D). *$p < 0.05$.

Table S1

Antibodies		
**Name**	**Company**	**Cat. No**
p16	Abcam	ab108349
p16	CST	23200s
Pcna	Abcam	ab29
γH2AX	Abcam	ab812999
Hnf4α	Abcam	ab92378
β-Actin	Abclonal	Ac026
p21	Abclonal	A2619
Rpl3	Abclonal	A6973
Dhx9	Abclonal	A4563
Polr2a	Abclonal	A2107
Ubc9	CST	4786s
Sumo1	CST	4930s
Fasn	CST	3180s
p-HSL	CST	4126s
HSL	CST	4170s
HA	CST	3724s
Flag	CST	2368s
Ubiqutin	CST	3936s
eIF2α	CST	5324s

p-eIF2α	CST	9721s
p53	Proteintech	60283-2-Ig
Lamin B	Proteintech	12987-1-AP
Cpt1a	Proteintech	15184-1-AP
Sirt1	Santa Cruz	Sc-15404
p-4E-BP1	Santa Cruz	Sc-293124
4E-BP1	Santa Cruz	Sc-514073
Alb	Santa cruz	Sc-374670
F4/80	Santa Cruz	Sc-377009
CD8	Santa Cruz	Sc-18913

Table S2

**List of primer sequences used for qPCR**		
Gene	Forward Sequence (5'-3')	Reverse Sequence (5'-3')
Rpl3	GGAAAGTGAAGAGCTTCCCTAAG	CTGTCAACTTCCCGGACGA
Actin	CATTGCTGACAGGATGCAGAAGG	TGCTGGAAGGTGGACAGTGAGG
P21	CCTGGTGATGTCCGACCTG	CCATGAGCGCATCGCAATC
P16	CGCAGGTTCTTGGTCACTGT	TGTTCACGAAAGCCAGAGCG
P53	CTCTCCCCCGCAAAAGAAAAA	CGGAACATCTCGAAGCGTTTA
Ki67	ATCATTGACCGCTCCTTTAGGT	GCTCGCCTTGATGGTTCCT
Pcna	TTTGAGGCACGCCTGATCC	GGAGACGTGAGACGAGTCCAT
Hnf4a	CACGCGGAGGTCAAGCTAC	CCCAGAGATGGGAGAGGTGAT

Fib	ATGCAACGATCAGGACACAA	TGTGCC TCT CAC ACTTCC AC
Col	CCTGGTAAAGAT GGTGCC	CACCAGGTTCACCTTTCG CACC
α-SMA	CGTACAACTGGTATTGTGCTGGAC	TGATGTCACGGACAATCTCACGCT
Tnf-α	CGTCGTAGCAAACCACCAAG	ATGAATCCAGGCATCGAAAAGC
IL-6	ATGGATGCTACCAAACTGGAT	TGAAGGACTCTGGCTTTGTCT
IL-4	CAACGAAGAACACCACAGAGAG	ATGAATCCAGGCATCGAAAAGC
IL-1β	GGATGAGGA CATGAGCACCT	TGAAGGACTCTGGCTTTGTCT
**List of primer sequences used for Chip-PCR**<br>**>NC_000070.7 Mus musculus strain C57BL/6J chromosome 4, GRCm39**		
Location	Forward Sequence (5'-3')	Reverse Sequence (5'-3')
P1(-2058/-1794)	GACTTGAGCTTCTCCACCTGACT	CCCTGTTGCCTGATGTCTATTC
P2(-1818/-1562)	AGAGAATAGACATCAGGCAACAGG	TACCTTGGGTTAGCTCCACAGA
P3(-1576/-1353)	AGCTAACCCAAGGTATGCACTG	GCCTGGAAATAACCAAACTAACC
P4(-1441/-1152)	GGGAAAGATGGAAAAGCAGC	GCCAGATGTGCAAGATGACG
P5(-1177/-931)	GTCCCACGTCATCTTGCACAT	CCGAGGGCCGAGTTGATC
P6(-999/-725)	GCGGAGCTAGGACTGACCAA	CAGCCCGAGGACGTTGTTTA
P7(-754/-478)	AAACCGAAAATAAACAACGTCCT	CCCTTGCTCCGAGTCCATCT
P8(-533/-281)	GCTGGCCTCGCCGATCT	ATCCCGCTCGCTTCGCT
P9(-296/-67)	GCGAAGCGAGCGGGATC	CAGCCCCTTCGGCGTTG
